# Asymmetric Michael addition reactions catalyzed by calix[4]thiourea cyclohexanediamine derivatives

**DOI:** 10.3762/bjoc.14.164

**Published:** 2018-07-25

**Authors:** Zheng-Yi Li, Hong-Xiao Tong, Yuan Chen, Hong-Kui Su, Tangxin Xiao, Xiao-Qiang Sun, Leyong Wang

**Affiliations:** 1 Jiangsu Province Key Laboratory of Fine Petrochemical Engineering, School of Petrochemical Engineering, Changzhou University, Changzhou 213164, China; 2 Key Laboratory of Mesoscopic Chemistry of MOE, School of Chemistry and Chemical Engineering, Nanjing University, Nanjing 210023, China

**Keywords:** asymmetric Michael addition reaction, calix[4]arene, cyclohexanediamine, thiourea

## Abstract

A number of upper rim-functionalized calix[4]thiourea cyclohexanediamine derivatives have been designed, synthesized and used as catalysts for enantioselective Michael addition reactions between nitroolefins and acetylacetone. The optimal catalyst **2** with a mono-thiourea group exhibited good performance in the presence of water/toluene (v/v = 1:2). Under the optimal reaction conditions, high yields of up to 99% and moderate to good enantioselectivities up to 94% ee were achieved. Detailed experiments clearly showed that the upper rim-functionalized hydrophobic calixarene scaffold played an important role in cooperation with the catalytic center to the good reactivities and enantioselectivities.

## Introduction

During the past decades, asymmetric organocatalysis has played an important role as a tool for the syntheses of chiral molecules under mild conditions [1–4]. Among these reactions, the asymmetric Michael reaction is a powerful strategy to construct versatile intermediates due to its synthetic convenience and good stereoselectivity [5–6]. Accordingly, different versions of this reaction have been extensively studied. Notably, the Michael addition reaction of 1,3-dicarbonyl compounds to conjugated nitroalkenes is very important for the synthesis of chiral nitro carbonyl compounds, such as bioactive agrochemicals and drugs [7–8]. Although great progress has been made in this research field, it is still need of further effort to synthesize new efficient chiral organic catalysts for this kind of Michael reactions.

The thiourea functional group has played a critical role in organocatalysis due to its ability in forming hydrogen bonds with substrates. This may lead to activated forms of the substrates allowing the corresponding reaction to occur [9–11]. For example, Jacobsen and co-workers pioneered an effective chiral thiourea catalyst which was employed in an asymmetric Strecker reaction [12–13]. In 2016, Hernández-Rodríguez and co-workers reported the preparation of bifunctional thioureas that contained either a methyl or trifluoromethyl group [14]. They discovered that the employment of chiral moieties with an α-trifluoromethyl group in thioureas show a positive effect on the selectivity and yields of the Michael reactions.

Supramolecular catalysis has drawn tremendous interest in the past few years [15–23]. In this context, calixarenes are ideal supramolecular macrocyclic scaffolds for the design of molecular receptors and organocatalysts due to their unique and tunable molecular architecture together with the ease of functionalization on the lower and upper rims [24–28]. Interestingly, their hydrophobic cavity also exhibits phase transfer catalytic function [29]. By attaching different pendants with catalytic ability to the scaffolds, this may offer us the opportunity to improve the green aspect of many reactions both in organic and aqueous medium [30]. For example, it has been reported that calixarenes linked with thiourea groups can be used to catalyze asymmetric Aldol reactions or Michael addition reactions in recent years [31–33]. Compared with the lower rim in the cone conformation of calixarenes, the functionalization of the upper rim is more challenging. Notably, it should be more valuable to exploit the cavity of upper rim-functionalized calixarenes because of the possibility of simultaneously using the hydrophobic cavity and chiral sites during a catalytic process [24–25].

Recently, we have reported a series of different functionalized organic catalysts based on calixarenes [26,34–38]. For example, we have been developed a calix[4]arene-based L-proline catalyst able to catalyze aldol reactions in aqueous solution with excellent enantioselectivity [35]. As part of our ongoing studies to develop novel types of organocatalysts for asymmetric catalysis, in this study, we aimed to synthesize novel upper rim-functionalized calix[4]thiourea cyclohexanediamine derivatives to catalyze asymmetric Michael addition reactions of acetylacetone and aromatic nitroalkenes.

## Results and Discussion

### Synthesis of catalysts

**Scheme 1:**
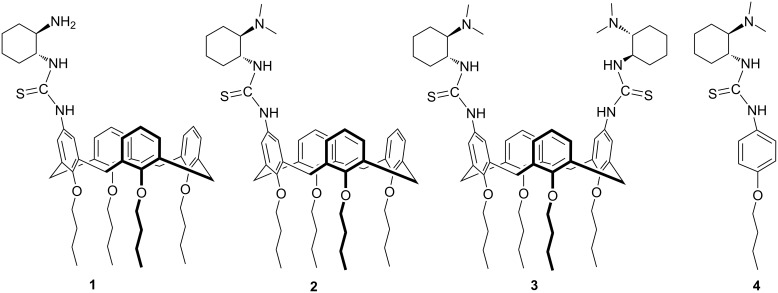
Catalysts synthesized and screened in this study.

**Scheme 2:**
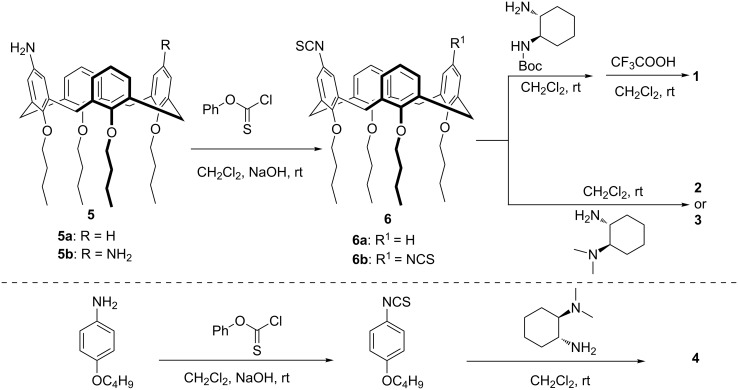
Synthetic routes for organocatalysts **1**–**4**.

The chemical structures and synthetic pathways for catalysts are shown in Scheme 1 and Scheme 2, respectively. A series of upper rim-functionalized calix[4]arene-based cyclohexanediamine derivatives **1**–**3** have been prepared. Calix[4]arene derivative **5** with an amino group on the upper rim was first prepared according to a literature report [38]. Then, the amino group was converted to an isothiocyano group through reaction with phenyl chlorothionocarbonate under alkaline conditions to obtain compound **6**. Subsequently, different chiral cyclohexanediamine derivatives were reacted with calix[4]arene-based compound **6** to form the corresponding substituted thioureas. By this route the monosubstituted primary amine **1**, monosubstituted tertiary amine **2** and disubstituted tertiary amine **3**, respectively, were obtained. Of note, for the preparation of compound **1** the chiral mono-Boc-protected cyclohexanediamine was used for the coupling reaction. The protecting group was removed by treatment with CF_3_COOH to afford **1**. Moreover, in order to comparatively study the role of the cavity of calix[4]arene, we also synthesized a model catalyst **4** by a similar synthetic procedure as outlined for catalyst **2**. All compounds **1**–**4** were fully characterized by NMR spectroscopy and HRMS analyses.

### Optimization of reaction conditions

Generally, conjugate additions were employed for evaluating the catalytic activities of the new chiral amino-substituted thioureas [33,39]. For this, the Michael addition reaction of acetylacetone (**8**) to β-nitrostyrene (**7a**) was chosen as the model reaction to evaluate the efficiency of compounds **1**–**4** as chiral organocatalysts (Table 1, entries 1–4). From Table 1, it can be seen that the yield with using the model catalyst **4** (only 75%) is significantly lower than those obtained with catalysts **1**–**3** in water (96–99%). Moreover, the reaction time using catalyst **4** was 6 h which is much longer compared to 1 h needed in case of catalysts **1**–**3**. These results showed that the catalysts comprising the calix[4]arene cavity are superior to the model catalyst in terms of catalytic reactivity. This is likely due to the formation of multiple microreactors at the water molecules’ interface, with the calix[4]arene hydrophobic cavity attracting reactants and accelerating the reaction [35,40]. The primary amine-containing catalyst **1** showed poor performance compared to the corresponding tertiary-amine containing catalysts **2** and **3**. Although catalysts **2** and **3** demonstrated similar reactivities, we chose monosubstituted catalyst **2** as catalyst for further optimization according to the principle of atomic economy.

**Table 1:** Screening of catalysts and solvents.^a^

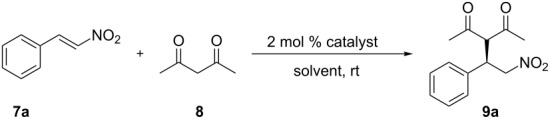					

entry	catalyst	solvent	time (h)	yield^b^ (%)	ee^c^ (%)

1	**1**	H_2_O	1	96	6
2	**2**	H_2_O	1	99	41
3	**3**	H_2_O	1	99	42
4	**4**	H_2_O	6	75	28
5	**2**	toluene	48	62	90
6	**2**	CH_3_CN	48	29	53
7	**2**	DMF	48	26	23
8	**2**	DMSO	48	23	23
9	**2**	1,4-dioxane	48	17	26
10	**2**	THF	48	56	77
11	**2**	CH_2_Cl_2_	48	37	58
12	**2**	Et_2_O	48	67	68
13	**2**	*n*-hexane	48	80	50
14	**2**	neat	48	13	70

^a^Reagents and conditions: catalyst (2 mol %), nitrostyrene (0.5 mmol), and acetylacetone (1 mmol), solvent (0.5 mL), rt; ^b^isolated yields; ^c^determined by chiral HPLC analysis.

Next, the effect of solvents on the reaction catalyzed by **2** was investigated and the results are summarized in Table 1. The results revealed that both the yield and the enantioselectivity were highly dependent on the solvents. Poor yields, lower enantioselectivities and long reaction time (48 h) were observed when the reactions were performed in organic solvents or without any solvent (Table 1, entries 6–14). Interestingly, using toluene as the solvent afforded a higher enantioselectivity (90% ee) with a low yield (62%, Table 1, entry 5), while H_2_O as the solvent gave higher yields (99% yield) with poor enantioselectivity (41% ee, Table 1, entry 2).

Therefore, in order to get good yield and enantioselectivity at the same time, a mixed solvent of toluene and water was chosen for the reaction (Table 2). The results showed that a good yield and enantioselectivity could be obtained when the volume ratio of toluene to water was 2:1 (Table 2, entry 5). We tried to further improve the catalytic effect by decreasing the reaction temperature and increasing the amount of acetylacetone. However, no improvements could be achieved (Table 2, entries 7 and 8). To our delight, increasing the catalyst loading of **2** from 2 mol % to 5 mol % resulted in a significant improvement in enantioselectivity (94% ee; Table 2, entry 9). However, further increasing the catalyst loading led to a slight decrease in enantioselectivity (Table 2, entry 10). Based on the above screening, the best results were obtained with 5 mol % of **2** in a mixed solvent of toluene and water (v/v = 2:1) at room temperature.

**Table 2:** Optimization of reaction conditions.^a^

entry	toluene/H_2_O (v/v)	time (h)	yield^b^ (%)	ee^c^ (%)

1	1:1	5	99	68
2	1:2	5	99	70
3	1:3	3	99	63
4	1:5	1	99	54
5	2:1	5	99	75
6	3:1	36	63	76
7^d^	2:1	40	47	74
8^e^	2:1	7	78	63
9^f^	2:1	4	99	94
10^g^	2:1	4	99	92

^a^Reagents and conditions: catalyst **2** (2 mol %), nitrostyrene (0.5 mmol), and acetylacetone (1 mmol), toluene and water (0.48 mL), rt; ^b^isolated yields; ^c^determined by chiral HPLC analysis; ^d^reaction performed at 0 °C; ^e^2.5 mmol acetylacetone used; ^f^5 mol % catalyst **2** used; ^g^10 mol % catalyst **2** used.

### The scope of reaction substrates

**Figure 1:**
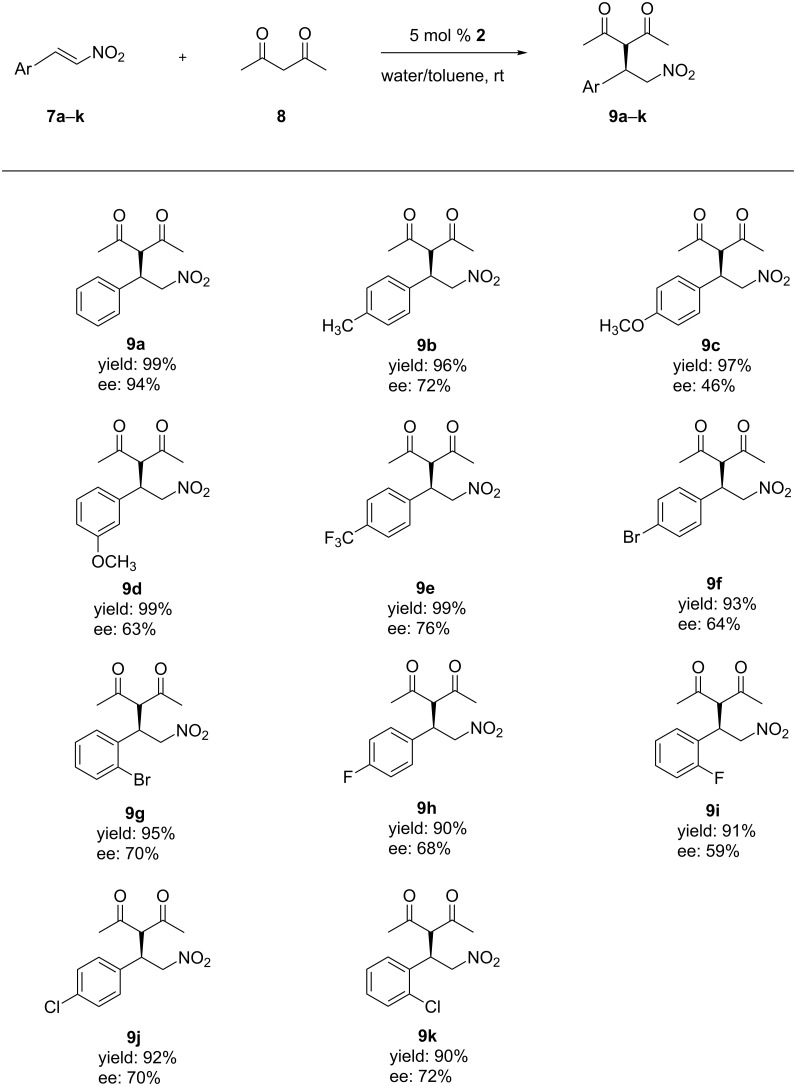
Asymmetric Michael addition of acetylacetone with different nitroolefins catalyzed by organocatalyst **2**. Reagents and conditions: catalyst **2** (5 mol %), nitroolefin (0.5 mmol), acetylacetone (1 mmol), toluene (0.32 mL) and water (0.16 mL), rt.

With the optimal reaction conditions in hand, a set of aryl nitroolefins **7a**–**k** was then employed to explore the generality of this protocol and the results are summarized in Figure 1. All nitroolefins reacted smoothly with acetylacetone (**8**) to afford the corresponding products **9a**–**k** in high yields (90–99%) and moderate enantioselectivities for **9b**–**k** (46–76% ee). In case of **9a** an excellent enantioselectivity (94% ee) was obtained. This might be due to the fact that the nitrostyrene **7a** lacking substituents has minimal steric hindrance and tends to bind with the calixarene cavity by supramolecular host–guest interactions which could further improve the enantioselectivity. In addition, electronic effects of the substituents on the aromatic ring showed a significant influence on the reaction. The presence of a strong electron-withdrawing trifluoromethyl group afforded the product **9e** (76% ee) with higher enantioselectivity than a strong-electron donating methoxy group (**9c**, 46% ee), while products with methyl and halogen groups showed moderate enantioselectivities (59–72% ee). For the same substituent at different positions of the aromatic ring, it can be seen that in case of the electron-donating methoxy group the position of the substituent has a remarkable effect on the enantioselectivity (*meta*: 63% ee, *para*: 46% ee). However, no obvious trends could be observed in case of *ortho-* or *para*-halogenated substrates.

### Mechanism study

**Scheme 3:**
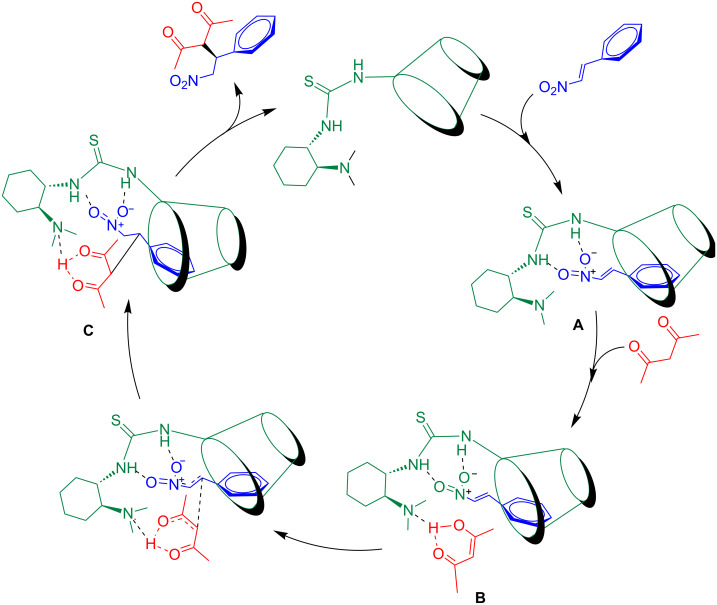
Possible proposed reaction mechanism.

There are two possibilities for the bifunctional thiourea-catalyzed asymmetric Michael addition reaction mechanism as has been summarized by Wang and co-worker [41]. In case of 1,3-dicarbonyl compounds or nitroolefins as substrates in the reaction, the question arises, which one is activated by the thiourea group through double hydrogen bonding. Based on the better enantioselectivity observed for product **9a** over **9b**–**k**, it was deduced that the binding of the nitroolefin with the calixarene cavity might be affected by the steric hindrance of the groups present on the aromatic ring. We propose the following plausible synergistic catalytic mechanism (Scheme 3). First, the two oxygen atoms of the nitro group in the nitrostyrene are activated through double hydrogen bonding with the thiourea group, while the benzene ring is held by a supramolecular host–guest interaction with the calixarene to form a stable transition state **A**. Then, another hydrogen bond is formed between the nitrogen atom of the tertiary amine group in **A** and acetylacetone in its enol form, leading to the formation of a ternary complex **B**. Finally, nucleophilic attack of acetylacetone on the nitrostyrene creates a new C–C bond forming binary complex **C** from which the enantioselective product is released.

## Conclusion

In summary, we have synthesized a series of upper rim-functionalized calix[4]arene-based chiral cyclohexanediamine thiourea catalysts **1**–**3** and tested as organocatalysts for the enantioselective Michael reactions of nitroolefins to 1,3-dicarbonyl compounds. Under the optimal conditions, catalyst **2** smoothly catalyzed the reactions in mixed solvent of toluene and water (v/v = 2:1) at room temperature to afford the products in high yields (90–99%) and with moderate to good enantioselectivities (46–94% ee). By comparing with model catalyst **4**, the calixarene scaffold, especially its hydrophobic cavity present in catalyst **2** played an important role in controlling reaction activities and enantioselectivities.

## Supporting Information

File 1 Experimental procedures, characterization data for all compounds and copies of NMR spectra.
