# Supplementary material for: Asymmetric Michael addition reactions catalyzed by calix[4]thiourea cyclohexanediamine derivatives
Source: Beilstein J Org Chem. 2018 Jul 25;14:1901–7. doi: 10.3762/bjoc.14.164 (PMC6071695; doi:10.3762/bjoc.14.164)
Supplement: File 1 — Experimental procedures, characterization data for all compounds and copies of NMR spectra. [file Beilstein_J_Org_Chem-14-1901-s001.pdf]

Supporting Information

for

Asymmetric Michael addition reactions catalyzed by calix[4]thiourea cyclohexanediamine derivatives

Zheng-Yi Li[1], Hong-Xiao Tong[1], Yuan Chen[1,2], Hong-Kui Su[1], Tangxin Xiao*[1], Xiao-Qiang Sun[1], and Leyong Wang*[1,2]

Address: [1]Jiangsu Province Key Laboratory of Fine Petrochemical Engineering, School of Petrochemical Engineering, Changzhou University, Changzhou 213164, China and [2]Key Laboratory of Mesoscopic Chemistry of MOE, School of Chemistry and Chemical Engineering, Nanjing University, Nanjing 210023, China

Email: Tangxin Xiao - xiaotangxin@cczu.edu.cn; Leyong Wang- lywang@nju.edu.cn

*Corresponding author

Experimental procedures, characterization data for all compounds and copies of NMR spectra

1. General....................S2
2. Synthesis of catalysts and characterization data....................S2
    2.1 Synthesis of isothiocyanato-calix[4]arenes....................S2
    2.2 Synthesis of catalyst **1**....................S3
    2.3 Synthesis of catalyst **2** and **3**....................S4
    2.4 Synthesis of catalyst **4**....................S5
3. Synthesis of substrates and characterization data....................S6
4. NMR spectra for catalysts and intermediates....................S11
5. NMR spectra for substrates....................S17
6. HPLC chromatograms....................S28
7. References....................S39

General

All chemicals were used as received without special purification unless stated otherwise. Analytical thin layer chromatography (TLC) was performed on precoated silica gel 60 F254 plates. Visualization on TLC was achieved by the use of UV light (254 nm). ^{1}H and ^{13}C NMR spectra were recorded on a Bruker 300 and 75 MHz NMR spectrometer using TMS as the internal standard. Melting points (mp) are determined with a MPA 100 apparatus and are not corrected. The ee values of products were determined by chiral-phase HPLC analysis. (1*R*, 2*R*)-*N*,*N*'-dimethylcyclohexane-1,2-diamine, (1*R*,2*R*)-*N*-Boc-cyclohexanediamine, **5a** and **5b** were prepared according to literature procedures [1,2].

2. Synthesis of catalysts and characterization data

2.1 Synthesis of isothiocyanato-calix[4]arenes

To a solution of **5a** or **5b** (1 equiv) in 20 mL DCM, NaOH (3 equiv or 6 equiv, respectively) was added and the resulting mixture was stirred at room temperature for 15 min. Next, phenyl chlorothionocarbonate (1 equiv or 2 equiv respectively) was added slowly in 5 min. After the reaction was complete, the reaction mixture washed with 10% HCl and deionized water. The aqueous layer was extracted with DCM, the combined organic phase was dried over MgSO4 and concentrated to give the crude product, which was purified by flash chromatography on silica gel (eluent with ethyl acetate/hexane 1:100) to afford product **6a** or **6b**.

Compound **6a**. White solid; 71% yield; mp: 116-117 ºC; ^{1}H NMR (300 MHz, $CDCl_3$): δ (ppm) = 0.95-1.02 (m, 12H, CH_3), 1.25-1.39 (m, 5H, CH_2), 1.47-1.59 (m, 3H, CH_2), 1.79-1.92 (m, 8H, CH_2), 3.08 (d, J = 13.5 Hz, 2H, ArCH_2Ar), 3.17 (d, J = 13.5 Hz, 2H, ArCH_2Ar), 3.75-3.80 (m, 4H, ArOCH_2), 3.87-4.03 (m, 4H, ArOCH_2), 4.39 (d, J = 9.0 Hz, 2H, ArCH_2Ar), 4.44 (d, J = 9.0 Hz, 2H, ArCH_2Ar), 6.19 (s, 2H, Ar*H*), 6.42 (d, J = 7.5 Hz, 2H, Ar*H*), 6.46-6.52 (m, 1H, Ar*H*), 6.78-6.95 (m, 6H, Ar*H*). ^{13}C NMR (75

MHz, $CDCl_3$): δ (ppm) = 13.9, 14.0, 14.1, 19.1, 19.4, 19.5, 30.8, 31.0, 32.1, 32.3, 32.4, 74.8, 74.9, 75.0, 77.2, 122.0, 122.2, 124.1, 124.9, 127.7, 128.4, 129.1, 134.0, 135.3, 136.0, 136.5, 155.4, 155.8, 157.3.

Compound **6b**. White solid; 75% yield; mp: 123–126 ºC; ^{1}H NMR (300 MHz, $CDCl_3$): δ (ppm) = 0.95-1.02 (m, 12H, CH_3), 1.34-1.41 (m, 4H, CH_2), 1.47-1.54 (m, 4H, CH_2), 1.81-1.92 (m, 8H, CH_2), 3.11 (d, J=13.5 Hz, 4H, ArCH_2Ar), 3.79-3.93 (m, 8H, ArOCH_2), 4.40 (d, J= 13.2 Hz, 4H, ArCH_2Ar), 6.38 (s, 4H, Ar*H*), 6.72-6.80 (m, 6H, Ar*H*). ^{13}C NMR (75 MHz, $CDCl_3$): δ (ppm) = 14.0, 14.1, 19.2, 19.4, 26.9, 29.7, 30.9, 32.1, 32.3, 75.0, 75.2, 77.2, 122.7, 124.7, 125.1, 128.7, 133.3 134.8, 136.3, 155.6, 156.6.

2.2 Synthesis of catalyst 1

To a solution of chiral (1*R*,2*R*)-*N*-Boc-cyclohexanediamine (0.21 mmol) in DCM (15 mL) was added **6a** (0.21 mmol). The reaction mixture was stirred for 0.5 h at room temperature. After removal of the solvent, the crude product was purified by flash chromatography on silica gel (eluent with ethyl acetate/hexane 1:4) to give Boc-protected product. Subsequently, CF_3COOH (1 mL) and DCM (30 mL) were added, and the mixture was stirred at room temperature for 3 h. After the reaction was complete, the solvent was distilled off. Then, DCM (30 mL) and H_2O (30 mL) were successively added, and 2.0 mol/L NaOH solution was added dropwise to adjust the pH to 8–9. The aqueous layer was extracted with DCM (3 × 20 mL), the combined organic phase was dried over $MgSO_4$ and concentrated to give the catalyst **1**.

Compound **1**. White solid; 89% yield; mp: 134-135 ºC; $[\alpha]_D^{20}$ -37.5° (C = 1.0, in $CHCl_3$); ^{1}H NMR (300MHz, DMSO-d_6): δ = 0.95-1.01 (m, 12H, CH_3), 1.22-1.53 (m, 14H, CH_2 + CH*H*CH_2CH_2CH*H*), 1.65-1.75 (m, 2H, C*H*HCH_2CH_2C*H*H), 1.82-2.02 (m, 10H, CH_2 + C*H*NH_2 + C*H*NH), 3.11-3.19 (m, 4H, ArCH_2Ar), 3.79-3.88 (m, 8H, ArOCH_2), 4.33-4.37 (m, 4H, ArCH_2Ar), 6.27-6.80 (m, 11H, Ar*H*), 7.56 (d, 1H, J = 8.4 Hz, N*H*), 7.88 (s, 2H, NH_2), 9.25 (s, 1H, N*H*). ^{13}C NMR (75MHz, DMSO-d_6): δ (ppm) = 14.3, 14.4, 19.3, 19.4, 23.7, 24.5, 29.9, 30.7, 31.4, 32.3, 53.5, 55.4, 74.8, 74.9, 75.0,

122.3, 128.2, 128.3, 128.5, 133.3, 134.6, 135.0, 135.2, 135.3, 154.6, 156.4, 156.6, 158.7, 159.2, 180.8. HR-ESI-MS ($C_{51}H_{69}N_3O_4S$): *m/z* calcd for $[M + H]^+$ = 819.5009, found = 819.5013.

2.3 Synthesis of catalyst 2 and 3

To a solution of chiral (1*R*,2*R*)-*N*,*N*'-dimethylcyclohexane-1,2-diamine (1.0 mmol) in DCM (15 mL) was added **6a** or **6b** (0.5 mmol). The reaction mixture was stirred for 0.5 h at room temperature. After removal of the solvent, the crude product was purified by flash chromatography on silica gel (eluent with CH_3OH/CH_2Cl_2 1:25) to afford product **2** or **3**.

Compound **2**. White solid; 81% yield; mp: 92–93 ºC; $[\alpha]_D^{20}$ -38.5° (*C* = 1.0, in $CHCl_3$); ^{1}H NMR (300MHz, DMSO-d_6): δ (ppm) = 0.95-0.99 (m, 12H, CH_3), 1.15-1.23 (m, 4H, $CH_2CH_2CH_2CH_2$), 1.34-1.73 (m, 12H, CH_2 + CH_2CH$_2$CH$_2$CH_2), 1.80-1.93 (m, 10H, CH_2 + C*H*N(CH$_3$)$_2$ + C*H*NH), 2.18 (S, 6H, N(CH_3)$_2$), 3.09-3.19 (m, 4H, ArCH_2Ar), 3.75-3.90 (m, 8H, ArOCH_2), 4.29-4.36 (m, 4H, ArCH_2Ar), 6.44-6.82 (m, 11H, Ar*H*), 7.13 (d, 1H, *J* = 6.6 Hz, N*H*), 9.11 (s, 1H, N*H*). ^{13}C NMR (75 MHz, DMSO-d_6): δ (ppm) = 14.3, 14.4, 19.3, 19.4, 21.8, 24.9, 25.1 30.6, 32.2, 32.3, 32.8, 55.3, 65.8, 74.7, 74.8, 74.9, 122.3, 123.1, 123.4, 128.1, 128.2, 128.3, 128.6, 128.7, 134.3,134.6, 134.7, 135.4, 135.5, 135.6, 153.5, 156.2, 156.8, 174.9. HR-ESI-MS ($C_{53}H_{73}N_3O_4S$): *m/z* calcd for $[M + H]^+$ = 847.5322, found = 847.5320.

Compound **3**. White solid; 55% yield; mp: 142–144 ºC; $[\alpha]_D^{20}$ -36.5° (*C* = 1.0, in $CHCl_3$); ^{1}H NMR (300 MHz, DMSO-d_6) δ (ppm) = 0.95-1.40 (m, 28*H*, CH_3 + CH_2CH$_2$CH_2CH_2) , 1.48-1.97 (m, 18H, CH_2 + C*H*N(CH$_3$)$_2$), 2.22 (s, 12H, N(CH_3)$_2$), 3.12 (d, 4H, *J* = 12.6 Hz, ArCH_2Ar), 3.70-3.97 (m, 10H, C*H*NH + ArOCH_2), 4.32 (d, 4H, *J* = 13.5 Hz, ArCH_2Ar), 6.36 (s 5H, Ar*H*), 7.00-7.23 (m, 5H, Ar*H*), 9.32 (s, 2H, N*H*). ^{13}C NMR (75 MHz, $CDCl_3$): δ (ppm) = 14.0, 14.1, 19.2, 19.5, 22.0, 24.6, 25.1, 29.7, 31.0, 31.1, 32.1, 32.4, 32.8, 40.2, 55.9, 66.7, 74.9, 75.0, 77.2, 122.3, 124.1, 128.8, 128.9, 135.1, 135.2, 135.8, 153.9, 157.3, 179.8. HR-ESI-MS ($C_{62}H_{90}N_6O_4S_2$): *m/z* calcd for $[M + H]^+$ = 1046.6465, found = 1046.6459.

2.4 Synthesis of catalyst 4

To a solution of 4-butoxyaniline (1.0 mmol) in 20 mL DCM, was added NaOH (4.5 mmol) and the resulting mixture was stirred at room temperature for 15 min. Next, phenyl chlorothionocarbonate was added slowly over 5 min. After the reaction was complete, the reaction mixture washed with 10% HCl (2 × 20 mL) and deionized water (2 × 20 mL). The aqueous layer was extracted with DCM (3 × 20 mL), the combined organic phase was dried over MgSO4 and concentrated to give the crude product, which was purified by flash chromatography on silica gel (eluent with ethyl acetate/hexane 1:100) to afford 1-butoxy-4-isothiocyanatobenzene. Then, 1-butoxy-4-isothiocyanatobenzene (0.5 mmol) was added to a solution of chiral (1*R*,2*R*)-*N*,*N*'-dimethylcyclohexane-1,2-diamine (1.0 mmol) in DCM (15 mL). The reaction mixture was stirred for 0.5 h at room temperature. After removal of the solvent, the crude product was purified by flash chromatography on silica gel (eluent with CH_3OH/CH_2Cl_2 1:20) to afford catalyst **4**.

Compound **4**. Yellow solid; 82% yield; $[\alpha]_D^{20}$ -27.6° (*C* = 1.0, in $CHCl_3$); ^{1}H NMR (300 MHz, DMSO-d_6): δ (ppm) = 0.93 (t, *J* = 7.5 Hz, 3H, CH_3), 1.04-1.24 (m, 4H, $CH_2CH_2CH_2CH_2$), 1.37-1.49 (m, 2H, CH_2), 1.55-1.78 (m, 5H, CH_2 + $CH_2CH_2CH_2CHH$), 2.21-2.31 (m, 7H, $N(CH_3)_2$ + $CH_2CH_2CH_2CHH$), 2.42-2.50 (m, 1H, $CHN(CH_3)_2$), 3.91-4.05 (m, 3H, $ArOCH_2$ + CHNH), 6.85-6.89 (m, 2H, Ar*H*), 7.21 (d, 1H, *J* = 6.9 Hz, N*H*), 7.26-7.29 (m, 2H, Ar*H*), 9.35 (s, 1H, N*H*). ^{13}C NMR (75 MHz, DMSO-d_6): δ (ppm) = 14.2, 19.2, 22.0, 25.0, 25.2, 31.3, 31.7, 32.7, 40.2, 55.3, 65.7, 67.7, 114.8, 125.4, 132.3, 156.0, 179.8. HR-ESI-MS ($C_{19}H_{31}N_3OS$): *m/z* calcd for $[M + H]^+$ = 349.2188, found = 349.2193.

3. Synthesis of substrates and characterization data

General procedure: To a stirred solution of the nitroalkene (0.5 mmol) and catalyst **2** (0.025 mmol, 5 mol %) in the mixed solvent of toluene (0.32 mL) and water (0.16 mL) was added acetylacetone (1 mmol). After the reaction was completed (monitored by TLC), the resulting mixture was concentrated and the residue was purified by flash

chromatography on silica gel (eluent with ethyl acetate/hexane 1:5 to 1:2) to afford the product. All products had NMR spectra in agreement with published data.

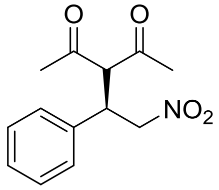

Compound **9a** [3]: (*R*)-3-(2-Nitro-1-phenylethyl)-pentane-2,4-dione

White solid; yield 99%; 94% *ee* determined by HPLC analysis (Daicel Chiralpak AS-H column, hexane/2-propanol = 85/15, flow rate: 1.0 mL/min, wavelength = 210 nm: t_R (minor) = 16.1 min, t_R (major) = 25.3 min). ^{1}H NMR (300 MHz, $CDCl_3$) δ (ppm) = 7.35-7.27 (m, 3H, C*H*), 7.20-7.17 (m, 2H, C*H*), 4.67-4.60 (m, 2H, CH_2), 4.37 (d, *J* = 10.8 Hz, 1H, C*H*), 4.28-4.20 (m, 1H, C*H*), 2.28 (s, 3H, CH_3), 1.94 (s, 3H, CH_3). ^{13}C NMR (75 MHz, $CDCl_3$) δ (ppm) = 29.7, 30.5, 42.8, 70.6, 78.2, 128.0, 128.5, 129.3, 136.0, 201.1, 201.8.

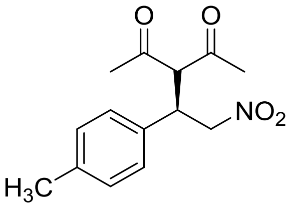

Compound **9b** [3]: (*R*)-3-(2-Nitro-1-(*p*-tolyl)ethyl)pentane-2,4-dione

White solid; yield 96%; 72% *ee* determined by HPLC analysis (Daicel Chiralpak AD-H column, hexane/2-propanol = 90/10, flow rate: 1.0 mL/min, wavelength = 210 nm: t_R (minor) = 10.9 min, t_R (major) = 17.3 min). ^{1}H NMR (300 MHz, $CDCl_3$): 7.14-7.05 (m, 4H, C*H*), 4.66-4.57 (m, 2H, CH_2), 4.36 (d, *J* = 10.8 Hz, 1H, C*H*), 4.24-4.14 (m, 1H, C*H*), 2.29 (s, 6H, CH_3), 1.94 (s, 3H, CH_3) ppm. ^{13}C NMR (75 MHz, $CDCl_3$) δ (ppm) = 201.9, 201.2, 138.4, 132.8, 130.0, 127.8, 78.4, 70.8, 42.5, 30.5, 29.5, 21.1.

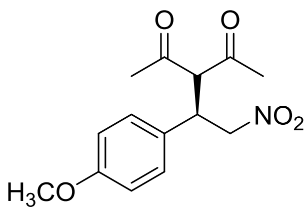

Compound **9c** [3]: (*R*)-3-[1-(4-Methoxyphenyl)-2-nitroethyl]pentane-2,4-dione

White solid; yield 97%; 46% *ee* determined by HPLC analysis (Daicel Chiralpak AD-H column, hexane/2-propanol = 90/10, flow rate: 1.0 mL/min, wavelength = 210 nm: t_R (minor) = 14.6 min, t_R (major) = 22.1 min). ^{1}H NMR (300 MHz, $CDCl_3$): 7.10 (d, *J* = 8.7 Hz, 2H, C*H*), 6.85 (d, *J* = 8.7 Hz, 2H, C*H*), 4.60-4.58 (m, 2H, CH_2), 4.33 (d, *J* = 11.1 Hz, 1H, C*H*), 4.23-4.15 (m, 1H, C*H*), 3.78 (s, 3H, OCH_3), 2.29 (s, 3H, CH_3), 1.94 (s, 3H, CH_3) ppm. ^{13}C NMR (75 MHz, $CDCl_3$) δ (ppm) = 201.9, 201.2, 159.5, 129.1, 127.6, 114.7, 77.4, 71.0, 55.2, 42.1, 30.4, 29.4.

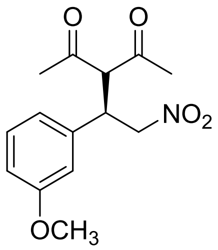

Compound **9d** [3]: (*R*)-3-[1-(3-Methoxyphenyl)-2-nitroethyl]pentane-2,4-dione

White solid; yield 99%; 63% *ee* determined by HPLC analysis (Daicel Chiralpak AD-H column, hexane/2-propanol =85/15, flow rate: 1.0 mL/min, wavelength = 210 nm: t_R (minor) = 9.6 min, t_R (major) = 12.3 min). ^{1}H NMR (300 MHz, $CDCl_3$): 7.23 (d, *J* = 8.1 Hz, 1H, C*H*), 6.83-6.71 (m, 3H, C*H*), 4.64-4.61 (m, 2H, CH_2), 4.37 (d, *J* = 10.8 Hz, 1H, C*H*), 4.25-4.20 (m, 1H, C*H*) 3.78 (s, 3H, OCH_3), 2.30 (s, 3H, CH_3), 1.97 (s, 3H, CH_3) ppm. ^{13}C NMR (75 MHz, $CDCl_3$) δ (ppm) = 201.8, 201.0, 160.1, 137.6, 130.4, 119.9, 114.2, 113.6, 78.2, 70.6, 55.3, 42.8, 30.5, 29.6.

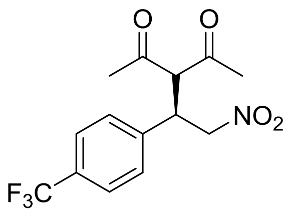

Compound **9e**[4]: (*R*)-3-[1-(4-Trifluoromethylphenyl)-2-nitroethyl]pentane-2,4-dione

Colorless oil; yield 99%; 76% *ee* determined by HPLC analysis (Daicel Chiralpak AD-H column, hexane/2-propanol = 90/10, flow rate: 1.0 mL/min, wavelength = 210 nm: t_R (minor) = 9.1 min, t_R (major) = 49.9 min). ^{1}H NMR (300 MHz, $CDCl_3$): 7.61 (d, J = 8.1 Hz, 2H, C*H*), 7.34 (d, J = 8.1 Hz, 2H, C*H*), 4.72-4.61 (m, 2H, CH_2), 4.41-4.29 (m, 1H, C*H*), 2.32 (s, 3H, CH_3), 2.00 (s, 3H, CH_3) ppm. ^{13}C NMR (75 MHz, $CDCl_3$) δ (ppm) = 201.2, 200.4, 140.3, 131.0, 130.6, 128.5, 126.3, 77.7, 70.3, 42.4, 30.5, 29.8.

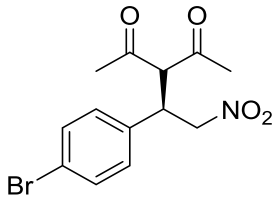

Compound **9f** [5]: (*R*)-3-[1-(4-Bromophenyl)-2-nitroethyl]pentane-2,4-dione

White solid; yield 93%; 64% *ee* determined by HPLC analysis (Daicel Chiralpak AD-H column, hexane/2-propanol = 85/15, flow rate: 0.7 mL/min, wavelength = 210 nm: t_R (minor) = 17.5 min, t_R (major) = 54.3 min). ^{1}H NMR (300 MHz, $CDCl_3$): 7.47 (d, J = 8.4 Hz, 2H, C*H*), 7.08 (d, J = 8.4 Hz, 2H, C*H*), 4.67-4.60 (m, 2H, CH_2), 4.33 (d, J = 10.8 Hz, 1H, C*H*), 4.26-4.18 (m, 1H, C*H*), 2.30 (s, 3H, CH_3), 1.98 (s, 3H, CH_3) ppm. ^{13}C NMR (75 MHz, $CDCl_3$) δ (ppm) = 201.4, 200.6, 135.1, 132.5, 129.7, 122.7, 77.9, 70.4, 42.2, 30.5, 29.7.

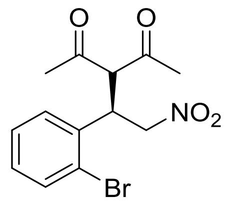

Compound **9g** [5]: (*R*)-3-[1-(2-Bromophenyl)-2-nitroethyl]pentane-2,4-dione

Orange solid; yield 95%; 70% *ee* determined by HPLC analysis (Daicel Chiralpak AD-H column, hexane/2-propanol = 98/2, flow rate: 0.7 mL/min, wavelength = 210 nm: t_R (minor) = 43.7 min, t_R (major) = 46.2 min). ^{1}H NMR (300 MHz, $CDCl_3$): 7.63 (d, *J* = 7.8 Hz, 1H, C*H*), 7.31-7.26 (m, 1H, C*H*), 7.20-7.13 (m, 2H, C*H*), 4.87-4.81 (m, 1H, C*H*), 4.78-4.65 (m, 2H, CH_2), 4.60 (d, *J* = 9.3 Hz, 1H, C*H*), 2.28 (s, 3H, CH_3), 2.05 (s, 3H, CH_3) ppm. ^{13}C NMR (75 MHz, $CDCl_3$) δ (ppm) = 202.0, 200.9, 135.1, 134.0, 130.0, 128.3, 77.5, 69.1, 41.1, 31.0, 28.5.

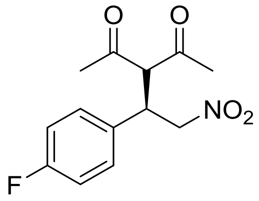

Compound **9h** [5]: (*R*)-3-[1-(4-Fluorophenyl)-2-nitroethyl]pentane-2,4-dione

Colorless oil; yield 90%; 68% *ee* determined by HPLC analysis (Daicel Chiralpak AD-H column, hexane/2-propanol = 90/10, flow rate: 0.7 mL/min, wavelength = 210 nm: t_R (minor) = 13.8 min, t_R (major) = 26.5 min). ^{1}H NMR (300 MHz, $CDCl_3$): 7.20-7.15 (m, 2H, C*H*), 7.06-6.99 (m, 2H, C*H*), 4.62-4.60 (m, 2H, CH_2), 4.36-4.20 (m, 2H, C*H*), 2.28 (s, 3H, CH_3), 1.96 (s, 3H, CH_3) ppm. ^{13}C NMR (75 MHz, $CDCl_3$) δ (ppm) = 201.5, 200.8, 164.2, 160.9, 131.8, 130.0, 129.5, 116.5, 116.3, 78.2, 70.7, 42.0, 30.5, 29.6.

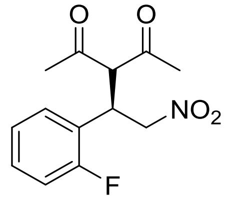

Compound **9i** [3]: (*R*)-3-[1-(2-Fluorophenyl)-2-nitroethyl]pentane-2,4-dione

Colorless oil; yield 91%; 59% *ee* determined by HPLC analysis (Daicel Chiralpak

AD-H column, hexane/2-propanol = 90/10, flow rate: 1.0 mL/min, wavelength = 210 nm: t_R(minor) = 10.5 min, t_R (major) = 12.0 min). ^{1}H NMR (300 MHz, $CDCl_3$): 7.33-7.26 (m, 1H, C*H*), 7.21-7.04 (m, 3H, C*H*), 4.80-4.61 (m, 2H, CH_2), 4.53-4.43 (m, 2H, C*H*), 2.29 (s, 3H, CH_3), 2.02 (s, 3H, CH_3) ppm. ^{13}C NMR (75 MHz, $CDCl_3$) δ (ppm) = 201.9, 200.9, 133.8, 133.5, 130.7, 129.7, 129.0, 127.7, 77.5, 68.9, 38.9, 30.9, 28.6.

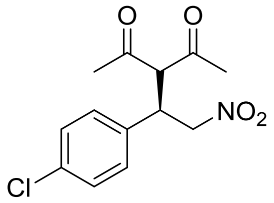

Compound **9j** [5]: (*R*)-3-[1-(4-Chlorophenyl)-2-nitroethyl]pentane-2,4-dione

White solid; yield 92%; 70% *ee* determined by HPLC analysis (Daicel Chiralpak AD-H column, hexane/2-propanol =85/15, flow rate: 0.7 mL/min, wavelength = 210 nm: t_R (minor) = 16.4 min, t_R (major) = 42.0 min). ^{1}H NMR (300 MHz, $CDCl_3$): 7.31 (d, *J* = 8.4 Hz, 2H, C*H*), 7.14 (d, *J* = 8.4 Hz, 2H, C*H*), 4.63-4.60 (m, 2H, CH_2), 4.34 (d, *J* = 10.5 Hz, 1H, C*H*), 4.27-4.19 (m, 1H, C*H*), 2.30 (s, 3H, CH_3), 1.98 (s, 3H, CH_3) ppm. ^{13}C NMR (75 MHz, $CDCl_3$) δ (ppm) = 201.4, 200.6, 134.6, 129.6, 129.3, 77.9, 70.5, 42.1, 30.5, 29.7.

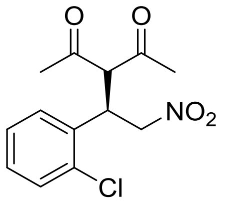

Compound **9k** [5]: (*R*)-3-[1-(2-Chlorophenyl)-2-nitroethyl]pentane-2,4-dione

White solid; yield 90%; 72% *ee* determined by HPLC analysis (Daicel Chiralpak AD-H column, hexane/2-propanol =98/2, flow rate: 0.7 mL/min, wavelength = 210 nm: t_R (minor) = 30.3 min, t_R (major) = 32.8 min). ^{1}H NMR (300 MHz, $CDCl_3$): 7.45-7.40 (m, 1H, C*H*), 7.26-7.21 (m, 2H, C*H*), 7.19-7.15 (m, 1H, C*H*), 4.87-4.58 (m, 4H, C*H* + CH_2), 2.28 (s, 3H, CH_3), 2.04 (s, 3H, CH_3) ppm. ^{13}C NMR (75 MHz, $CDCl_3$) δ (ppm) = 201.9, 200.9, 133.8, 133.5, 130.7, 129.7, 129.0, 127.7, 77.5, 68.9, 38.9, 30.9, 28.6.

4. NMR spectra for catalysts and intermediates

^{1}H NMR spectrum of **6a**

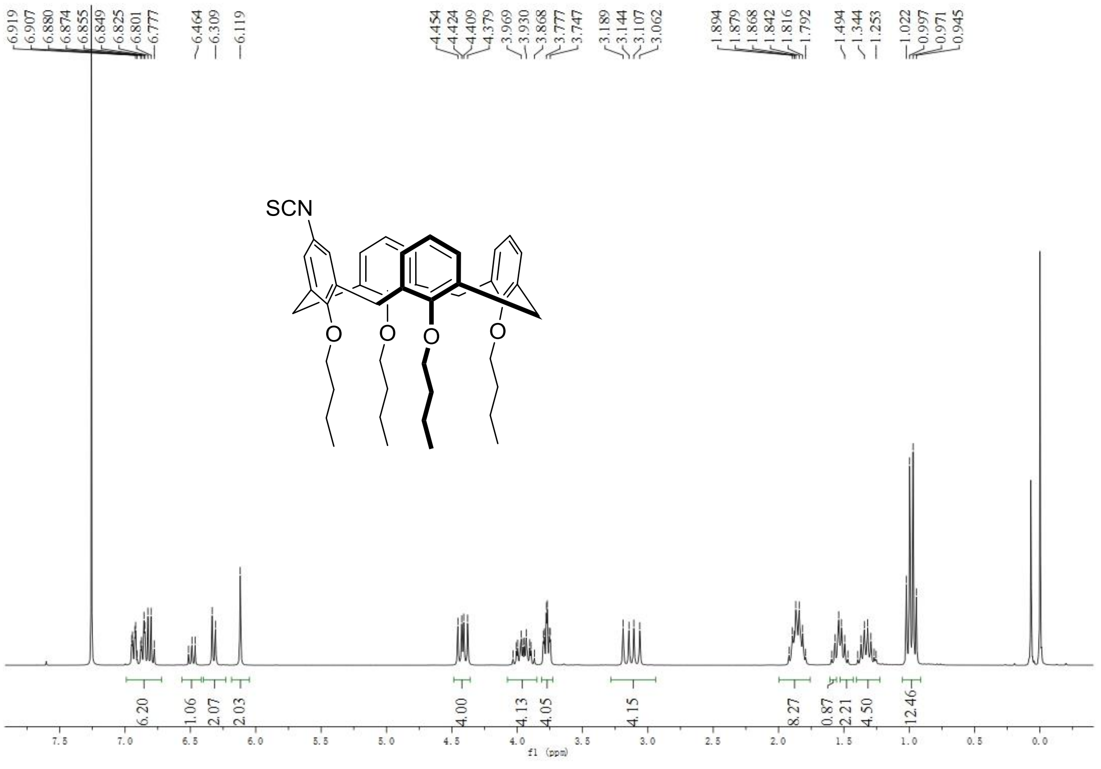

^{13}C NMR spectrum of **6a**

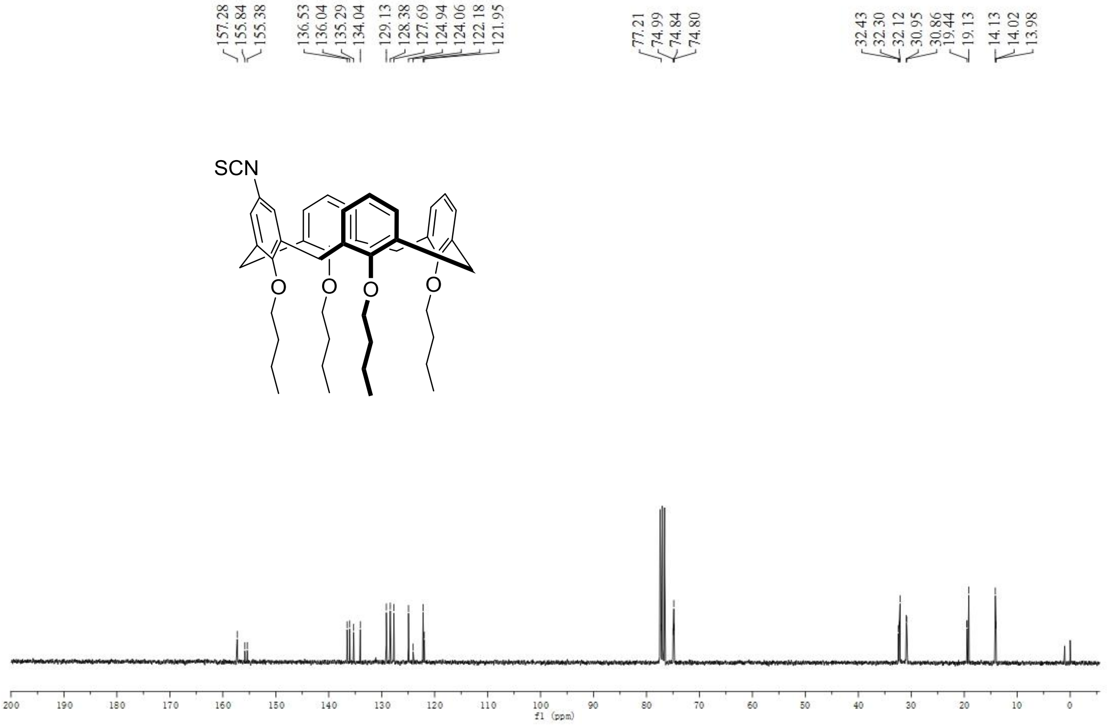

^{1}H NMR spectrum of **6b**

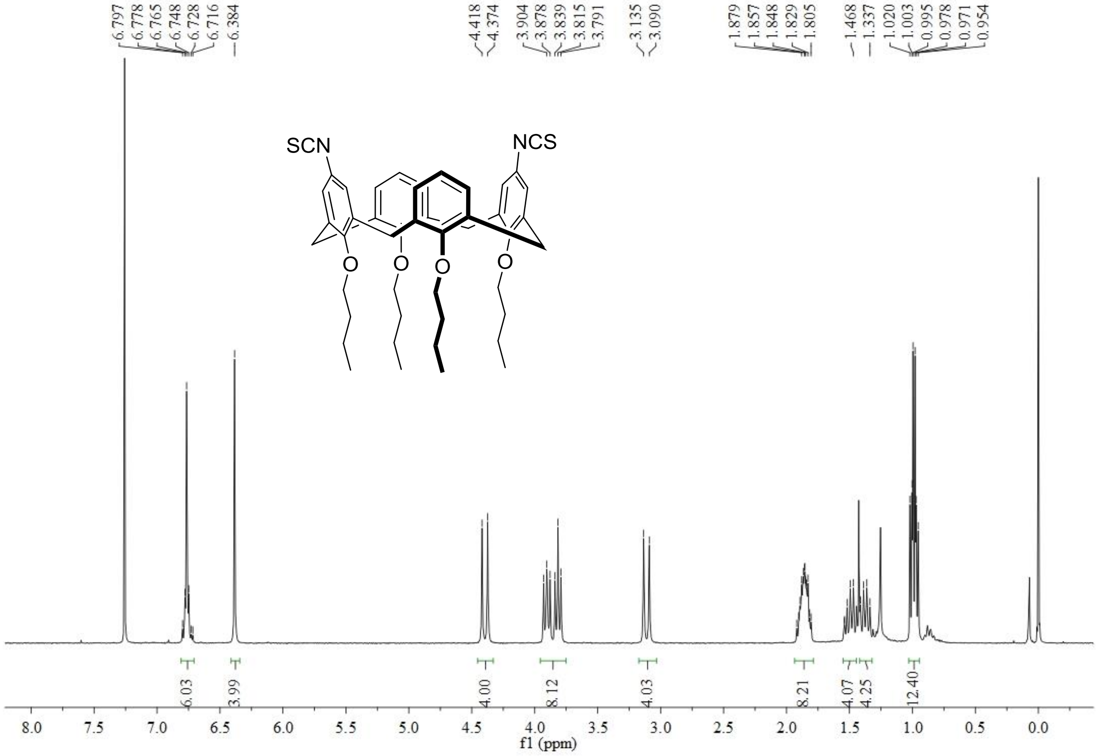

^{13}C NMR spectrum of **6b**

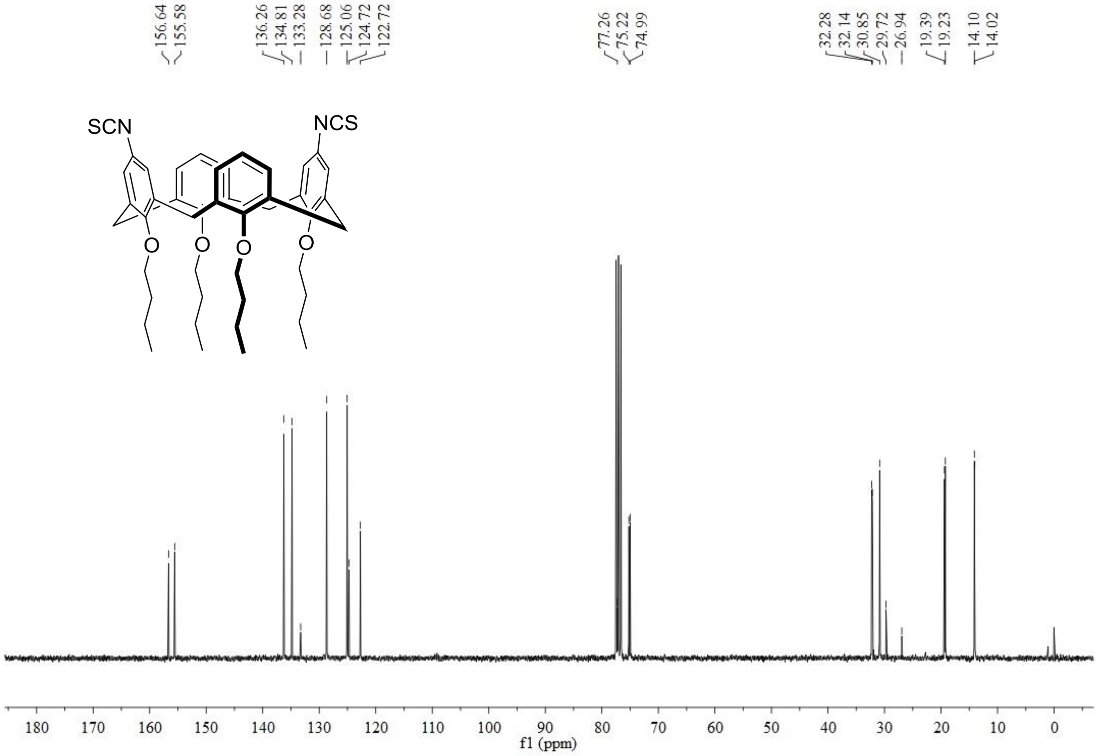

^{1}H NMR spectrum of catalyst **1**

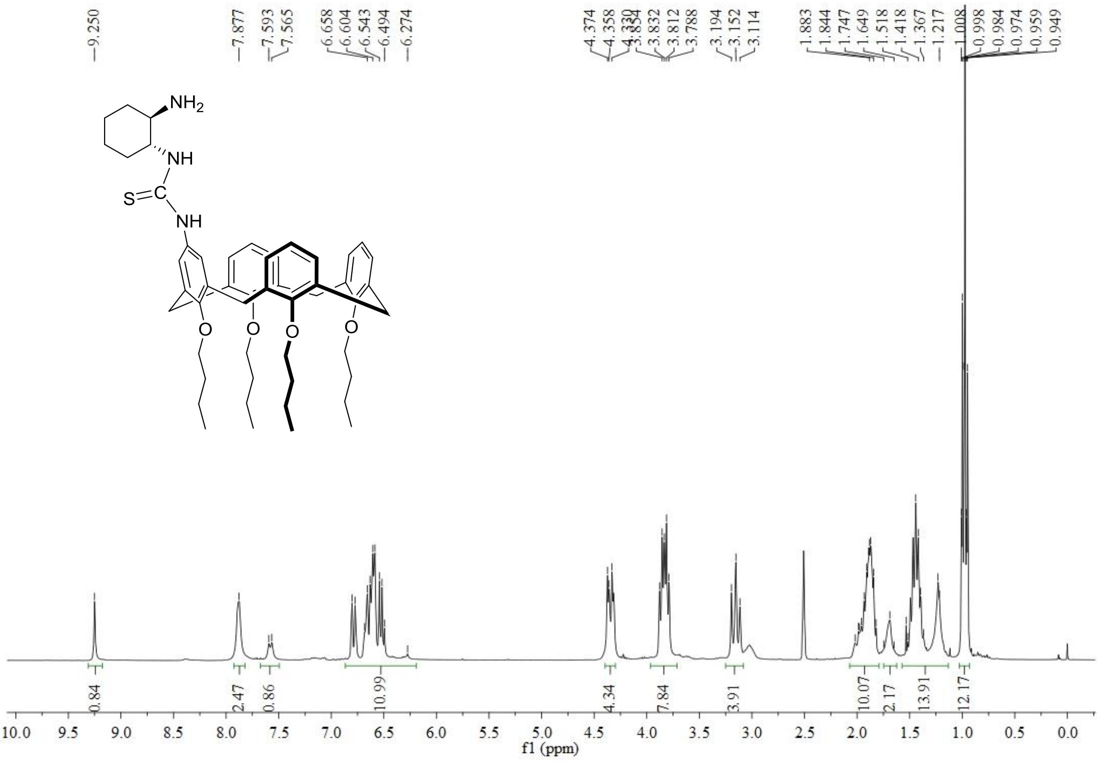

^{13}C NMR spectrum of catalyst **1**

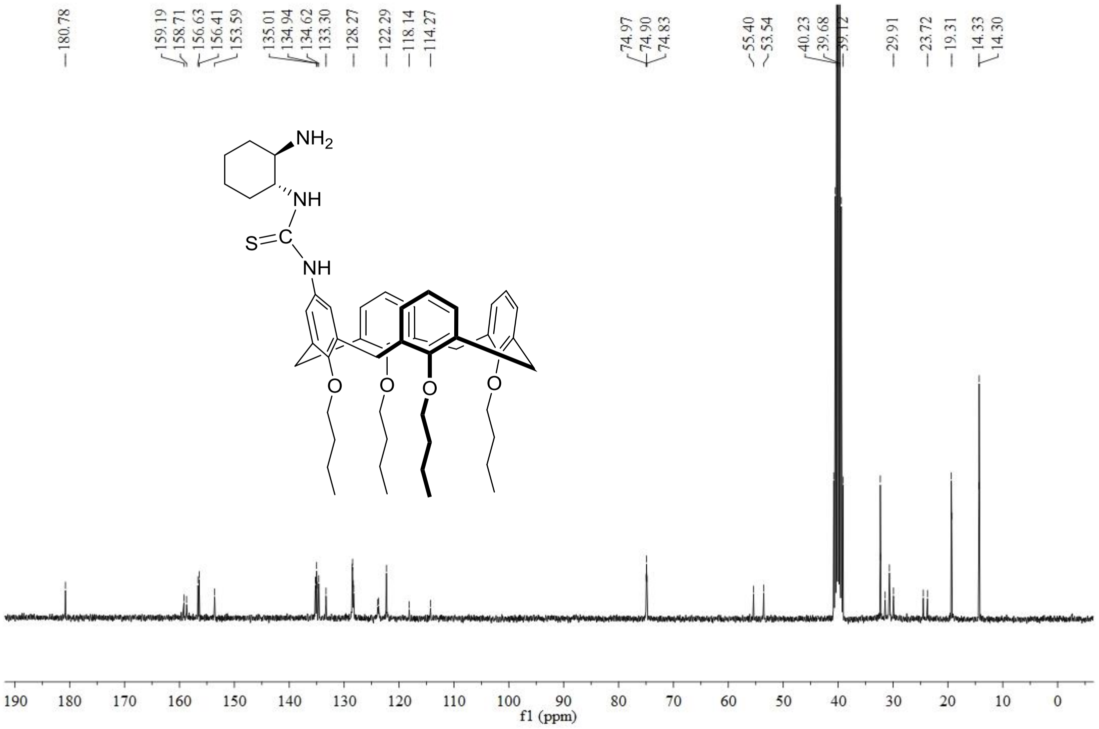

^{1}H NMR spectrum of catalyst **2**

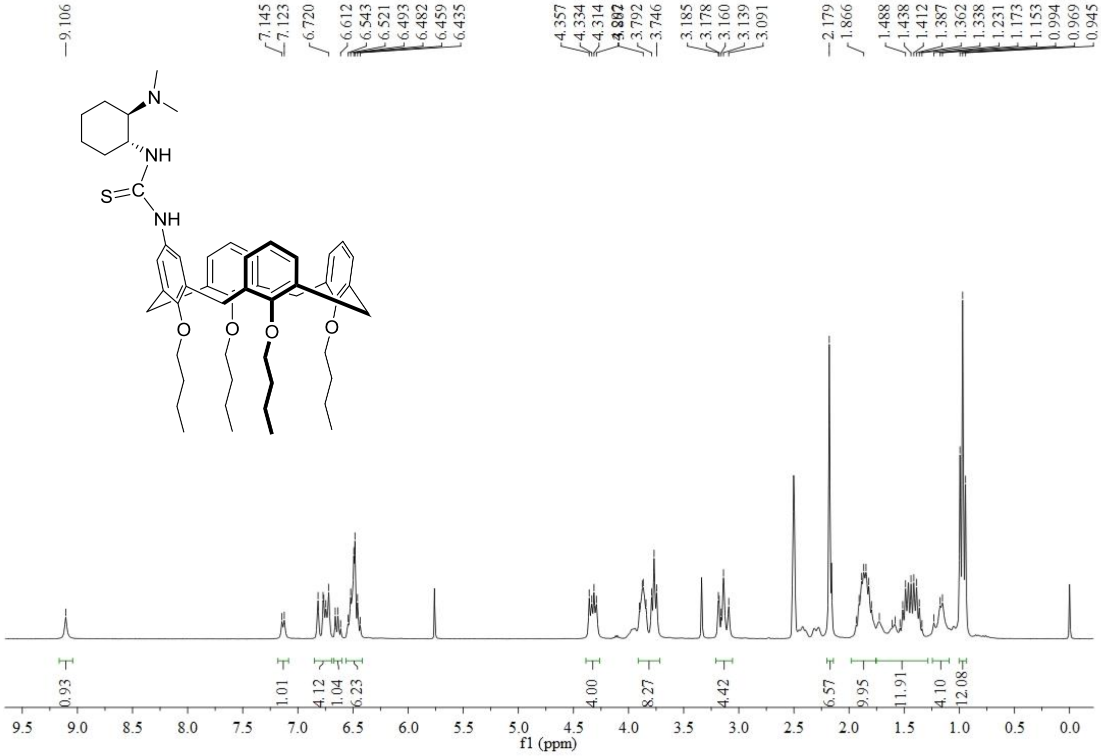

^{13}C NMR spectrum of catalyst **2**

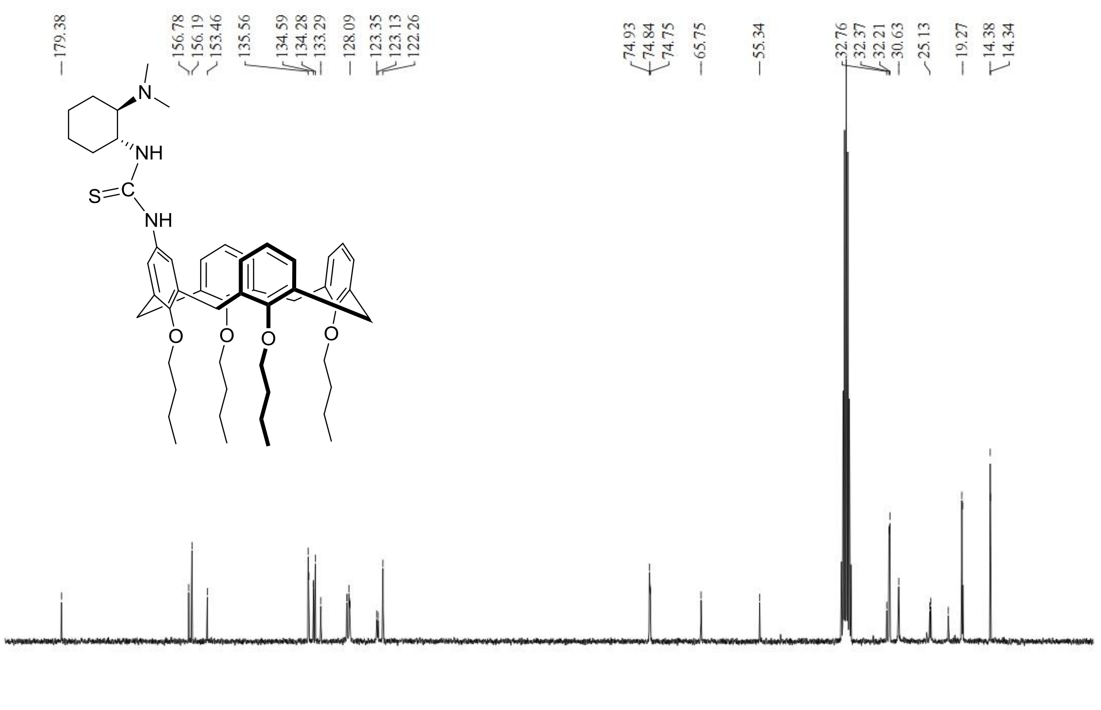

^{1}H NMR spectrum of catalyst **3**

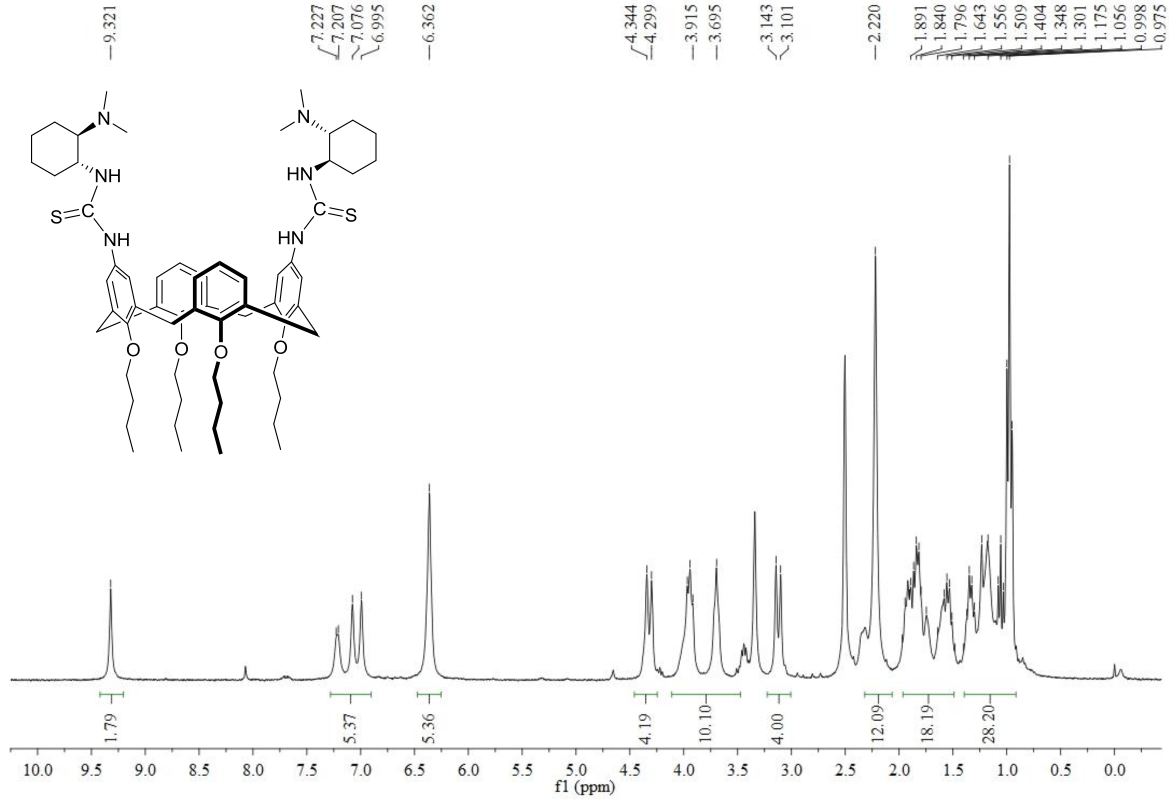

^{13}C NMR spectrum of catalyst **3**

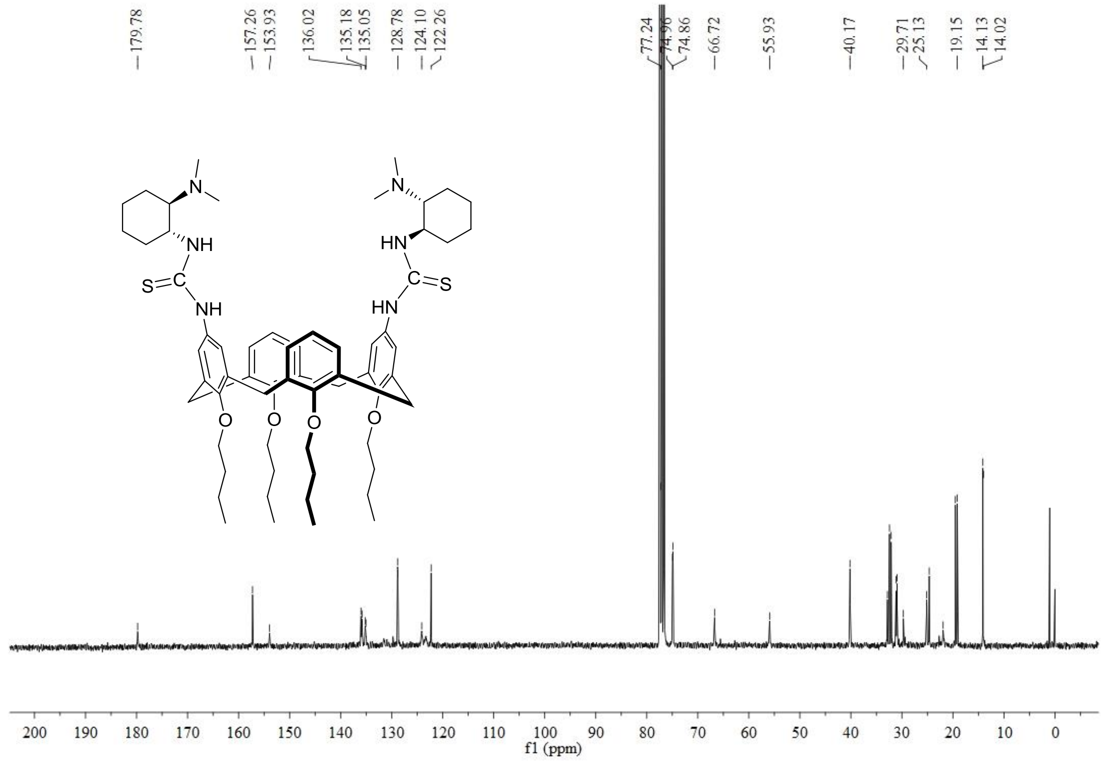

^{1}H NMR spectrum of catalyst **4**

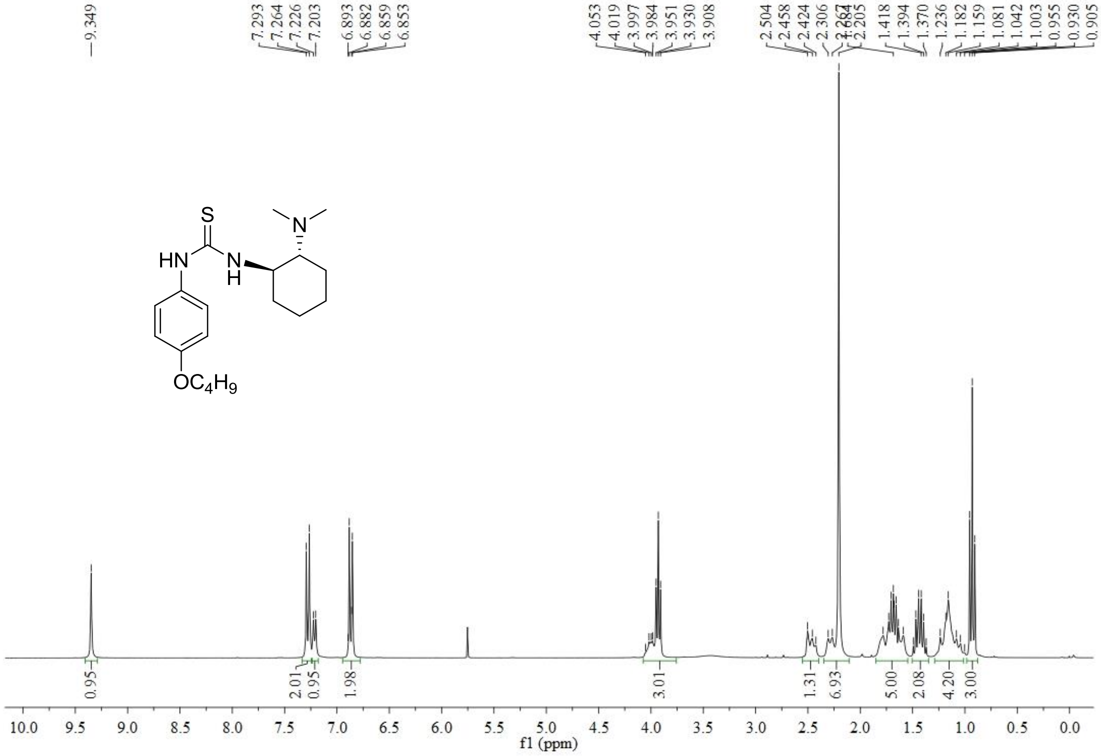

^{13}C NMR spectrum of catalyst **4**

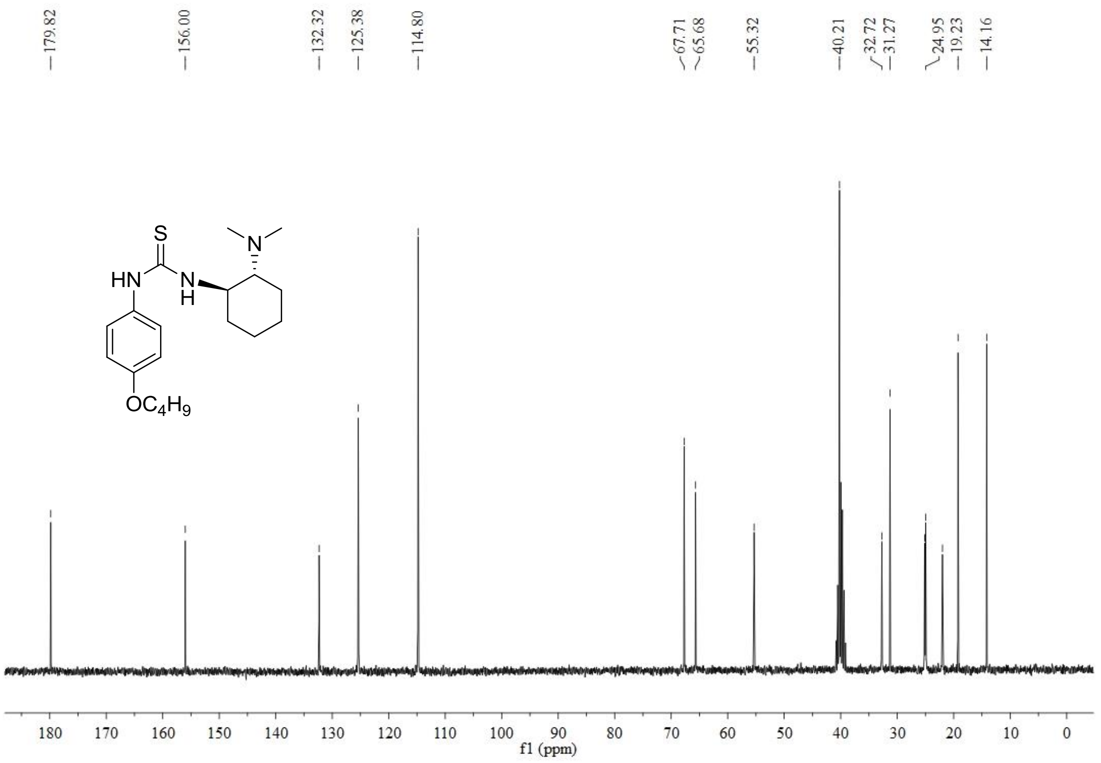

5. NMR spectra for products

^{1}H NMR spectrum of **9a**

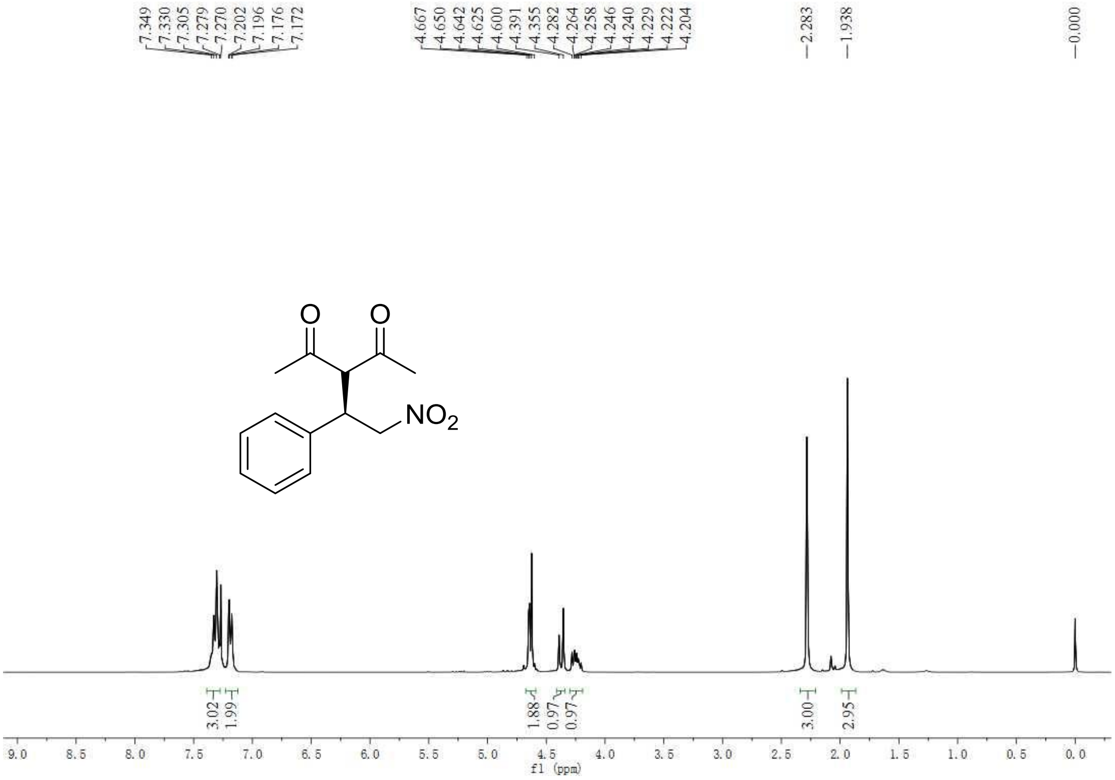

^{13}C NMR spectrum of **9a**

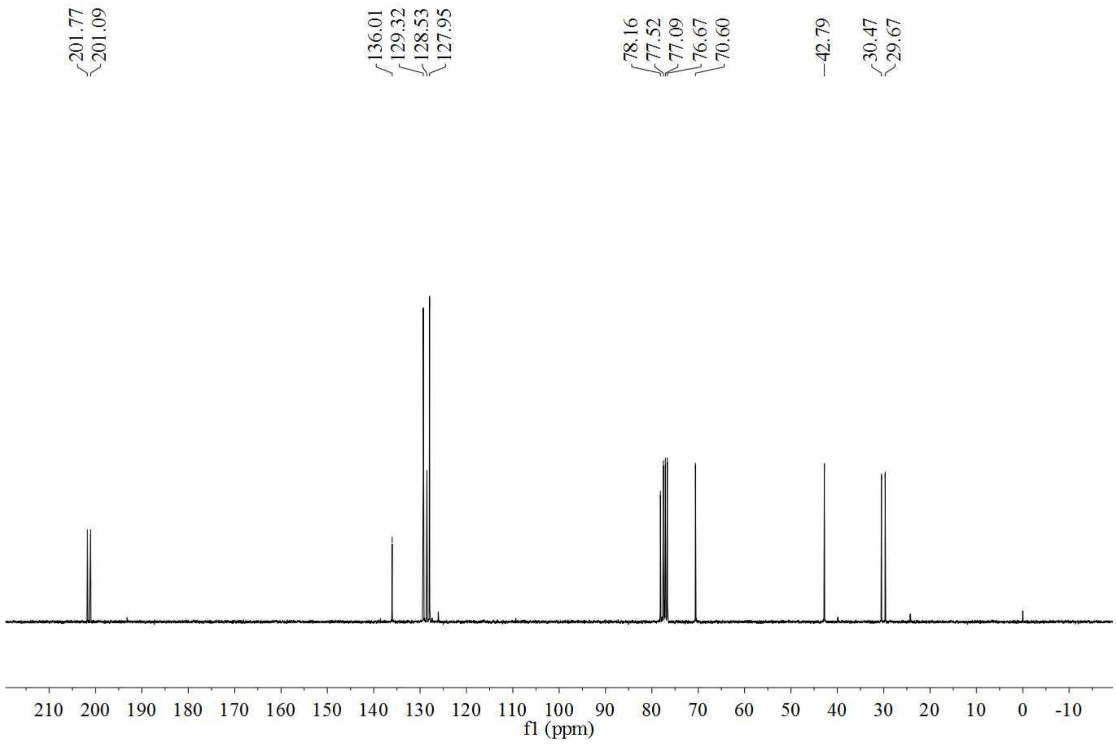

^{1}H NMR spectrum of **9b**

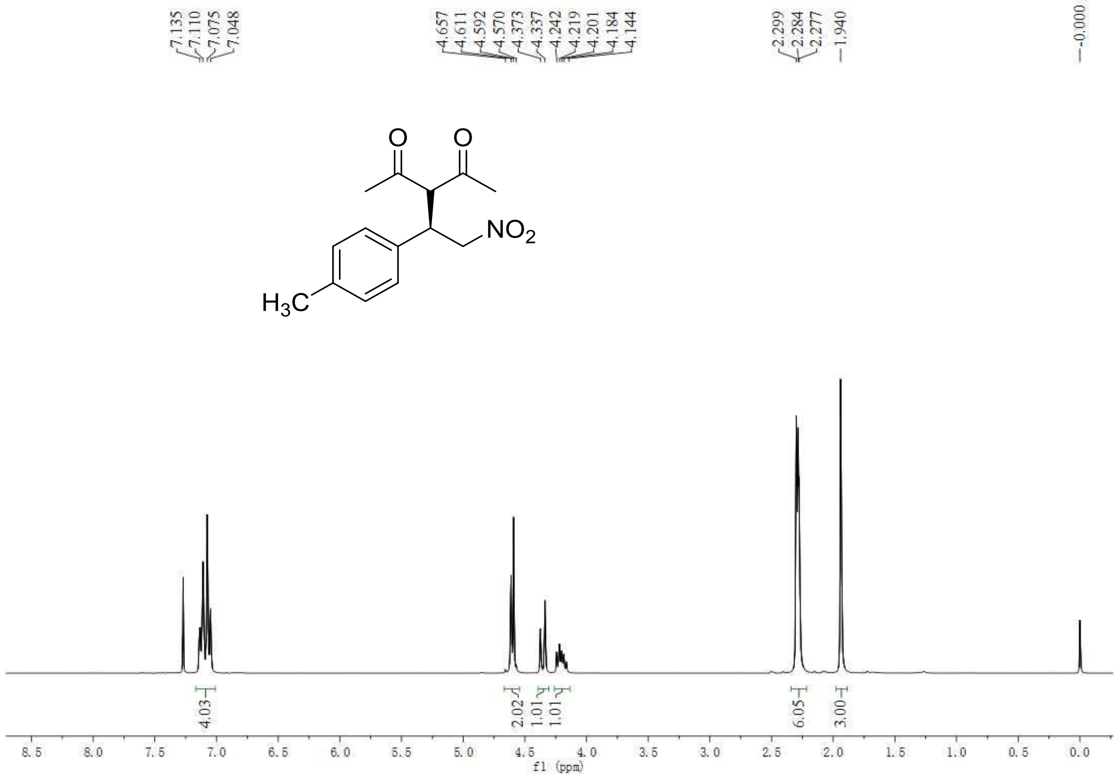

^{13}C NMR spectrum of **9b**

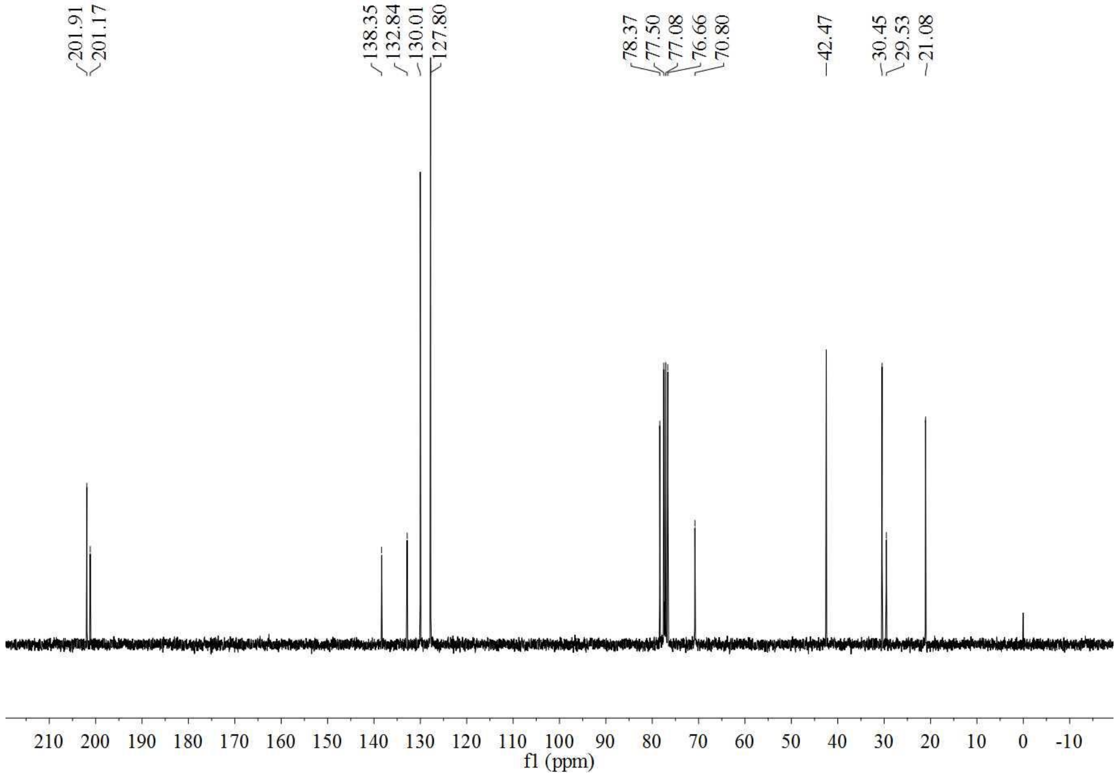

^{1}H NMR spectrum of **9c**

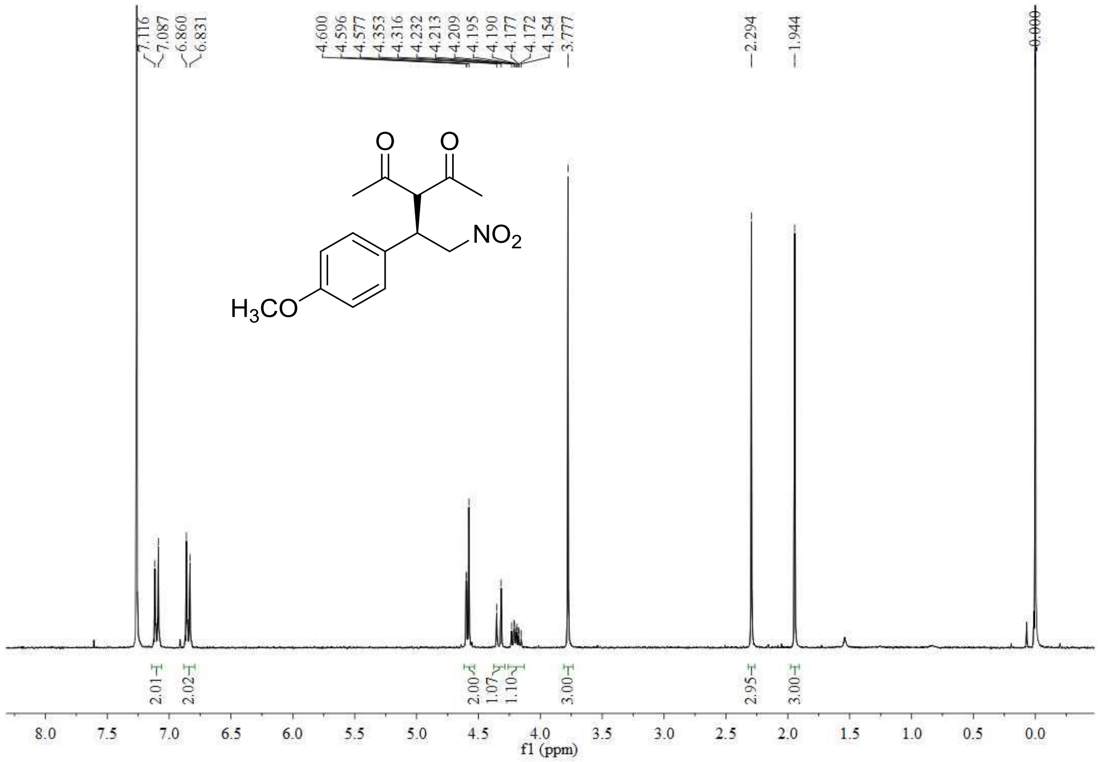

^{13}C NMR spectrum of **9c**

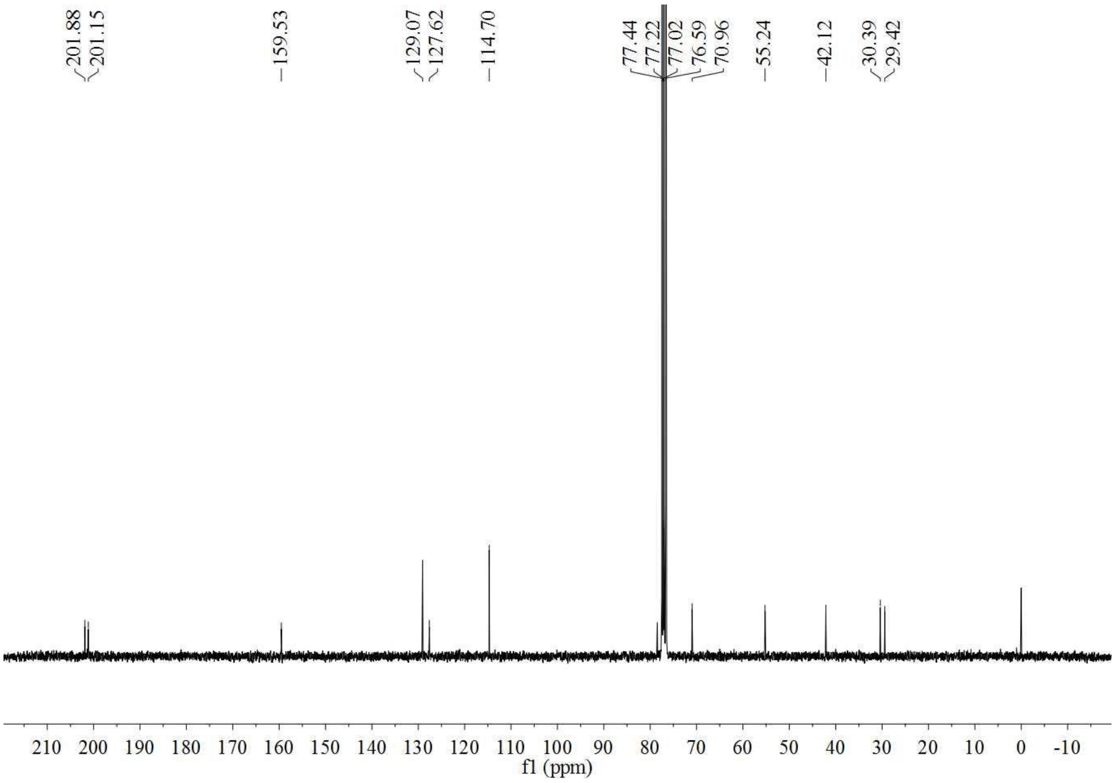

^{1}H NMR spectrum of **9d**

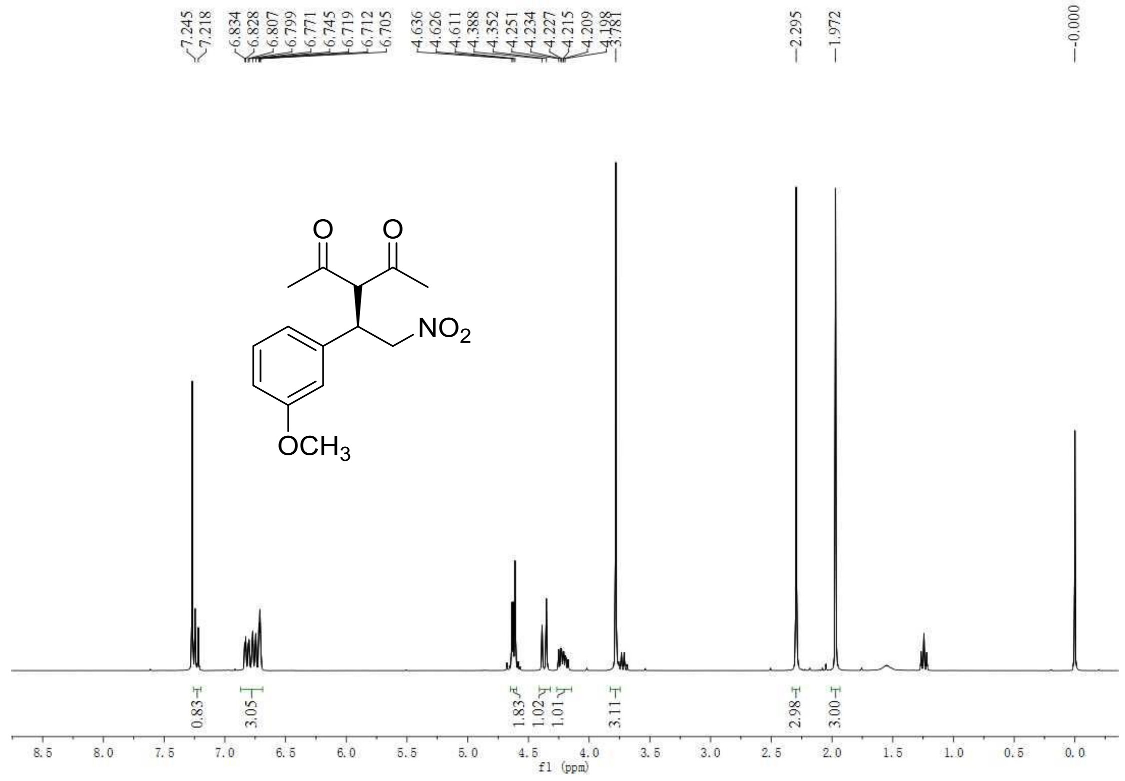

^{13}C NMR spectrum of **9d**

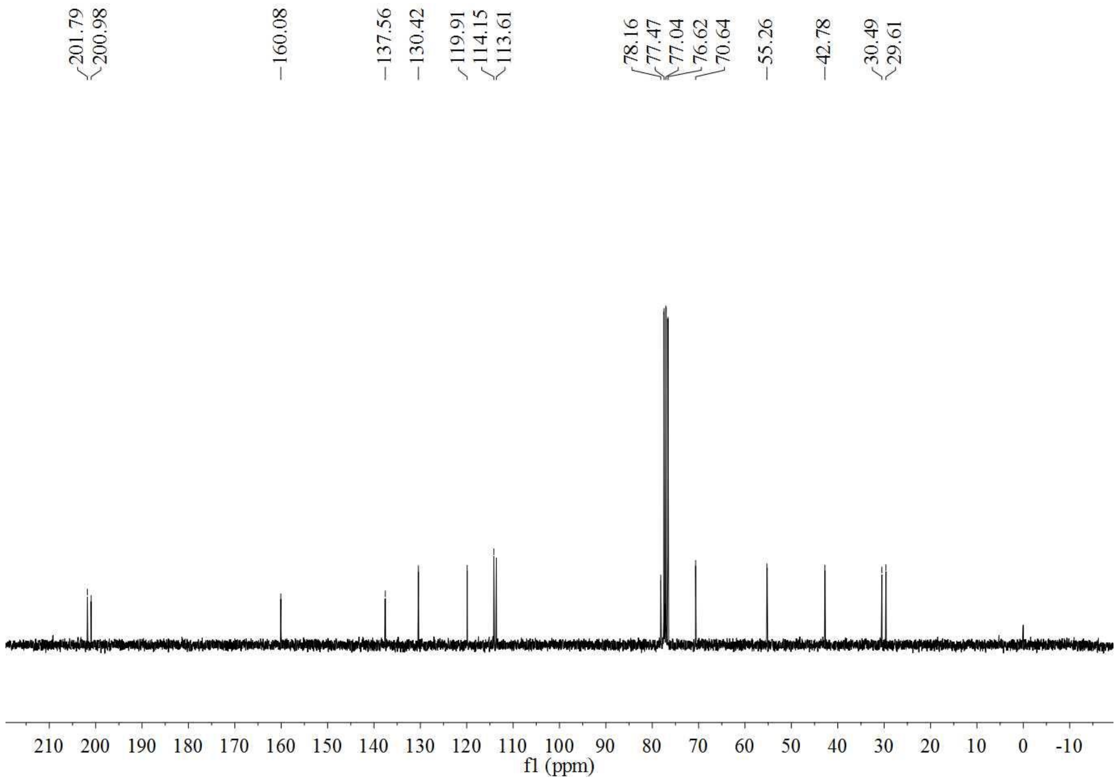

^{1}H NMR spectrum of **9e**

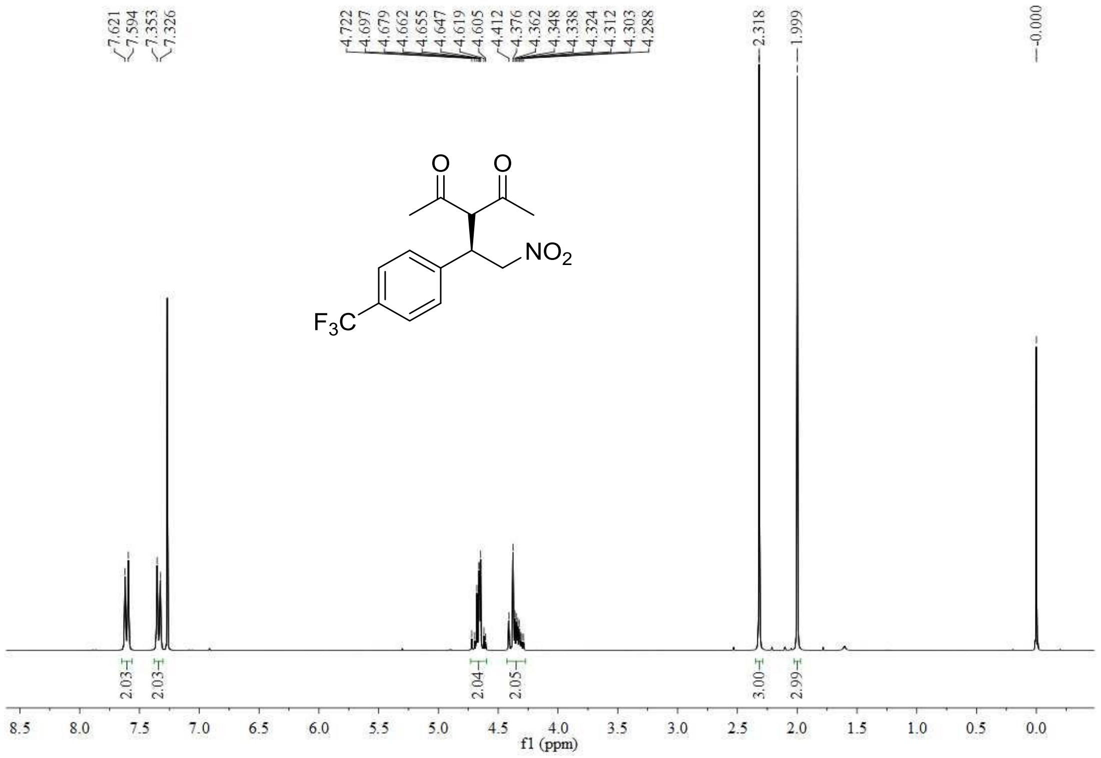

^{13}C NMR spectrum of **9e**

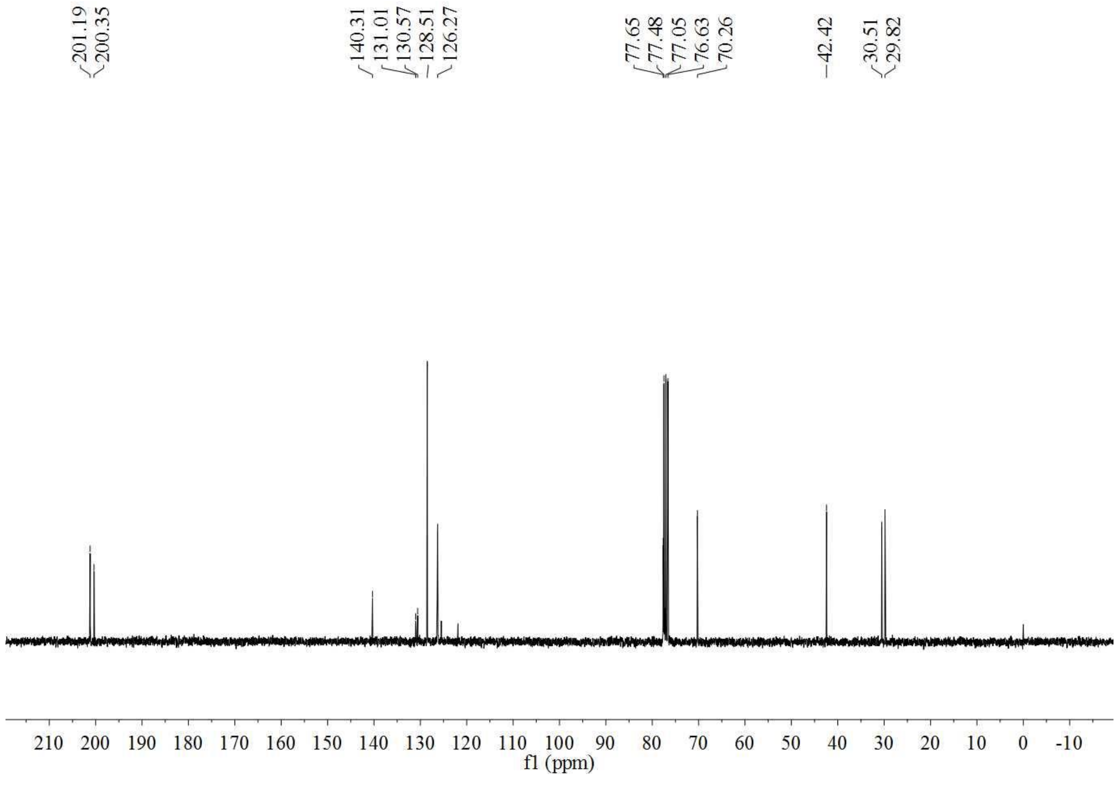

^{1}H NMR spectrum of **9f**

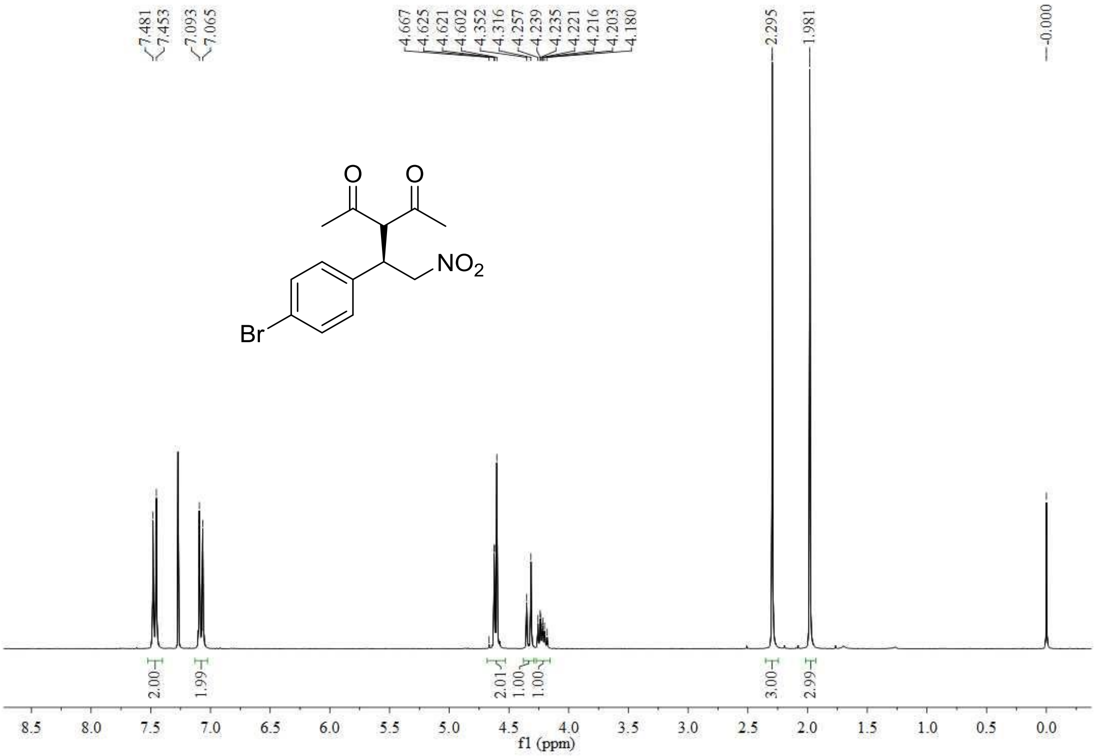

^{13}C NMR spectrum of **9f**

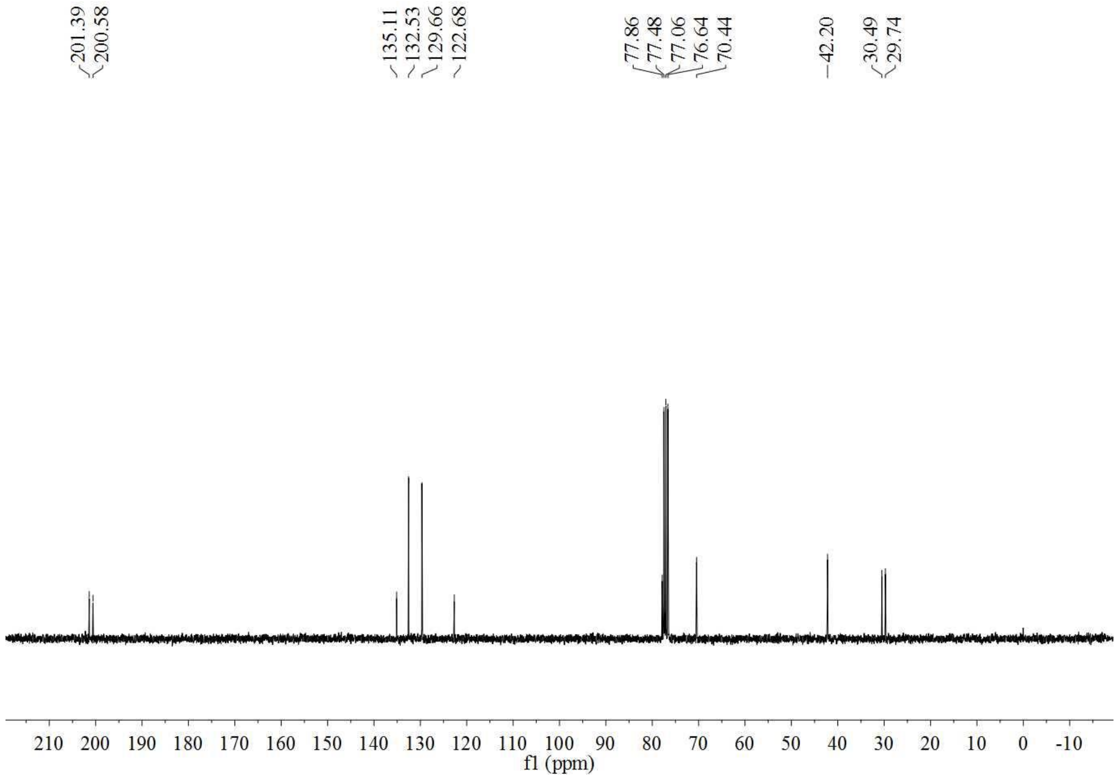

^{1}H NMR spectrum of **9g**

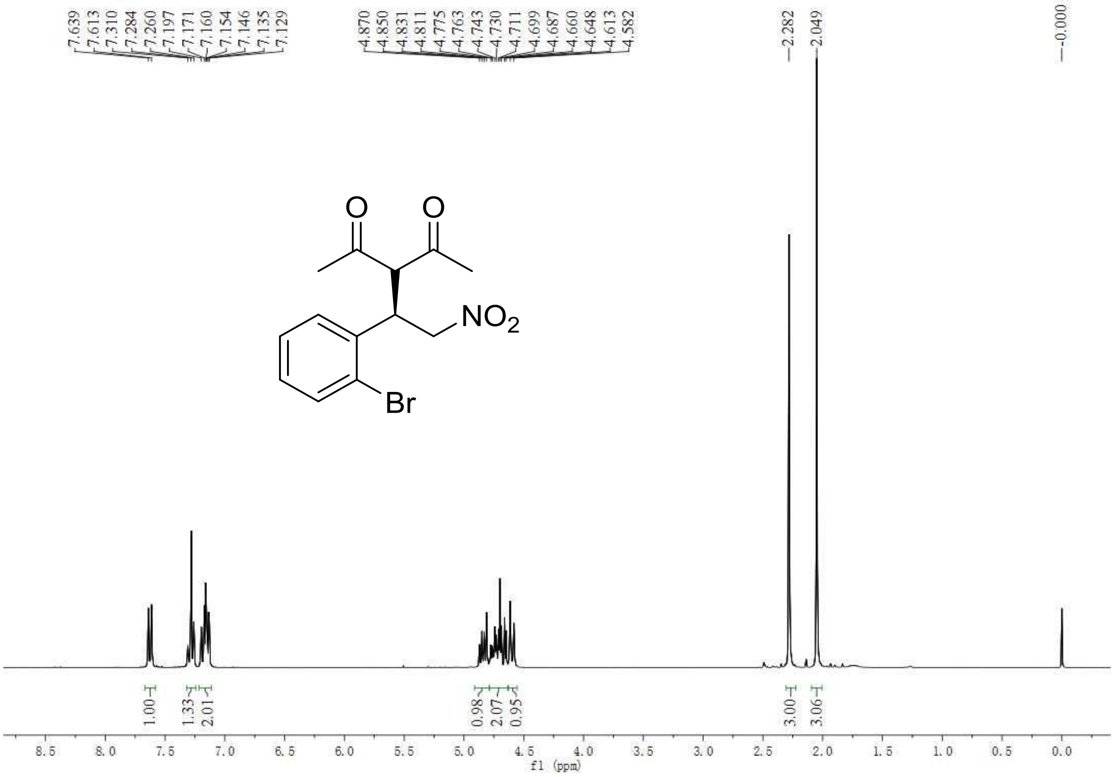

^{13}C NMR spectrum of **9g**

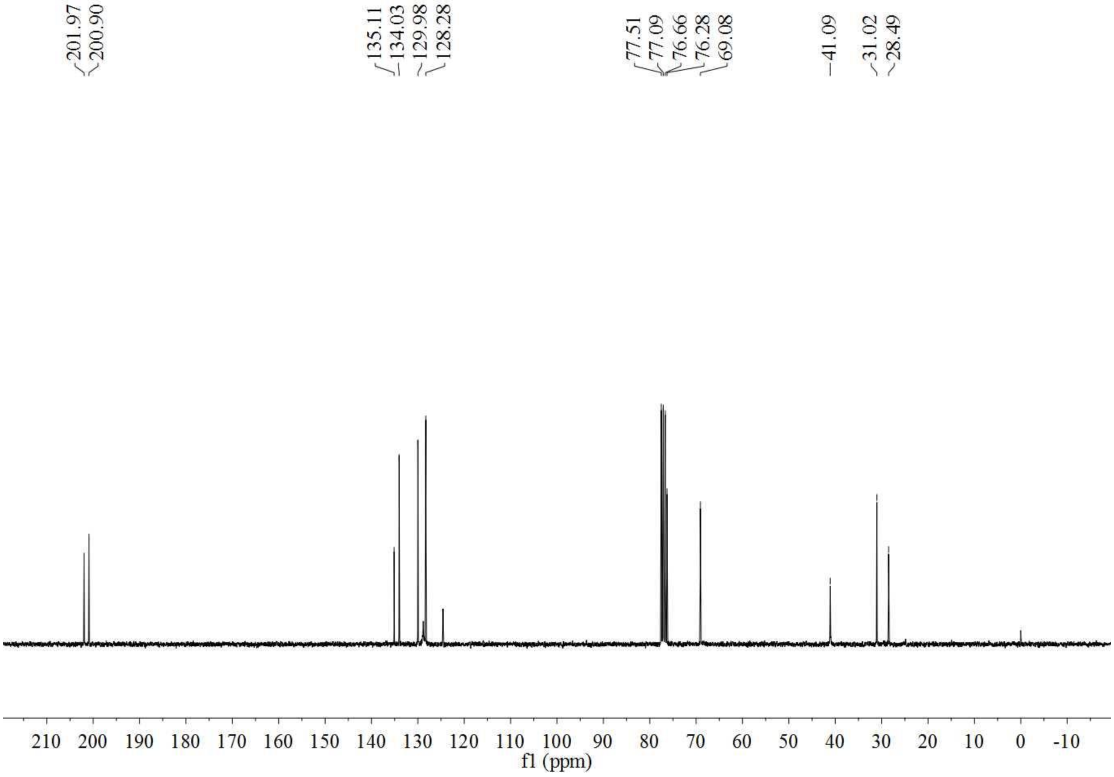

^{1}H NMR spectrum of **9h**

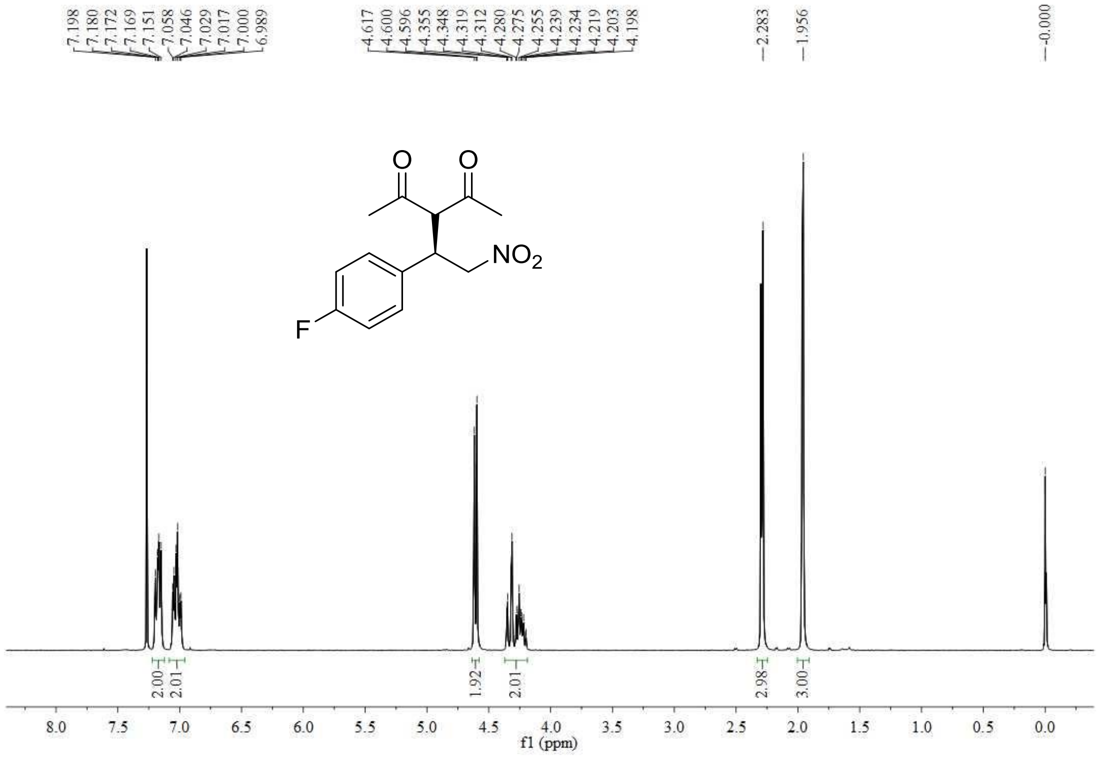

^{13}C NMR spectrum of **9h**

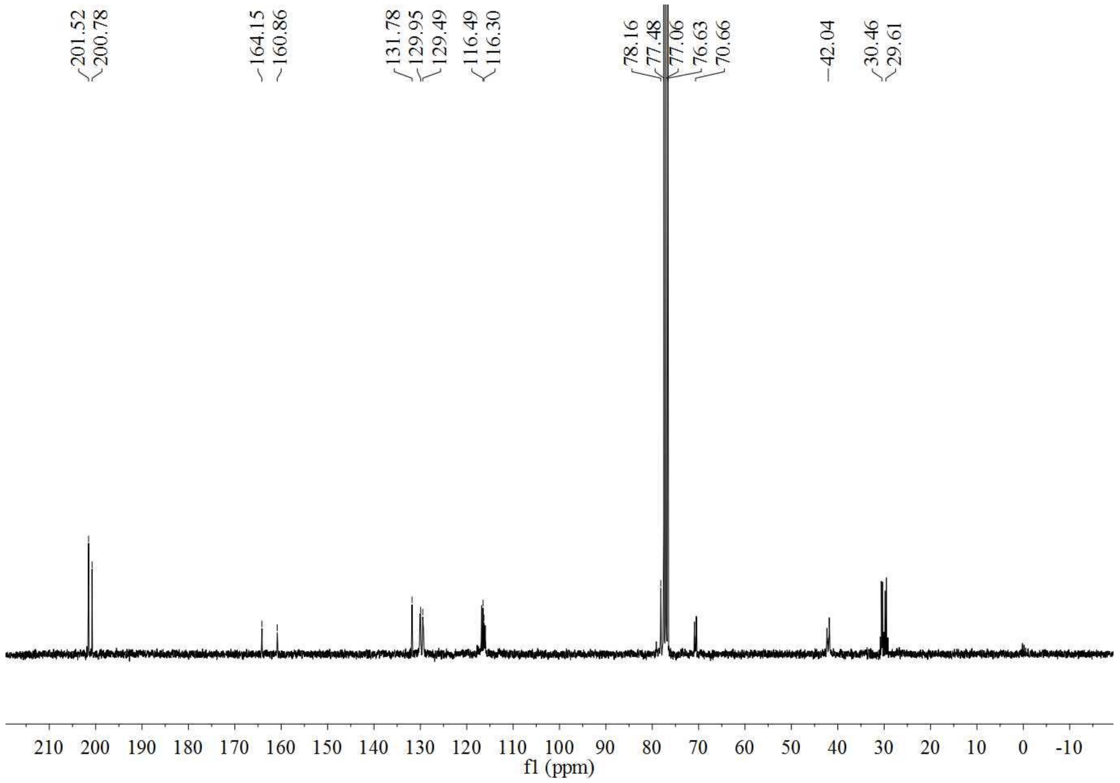

^{1}H NMR spectrum of **9i**

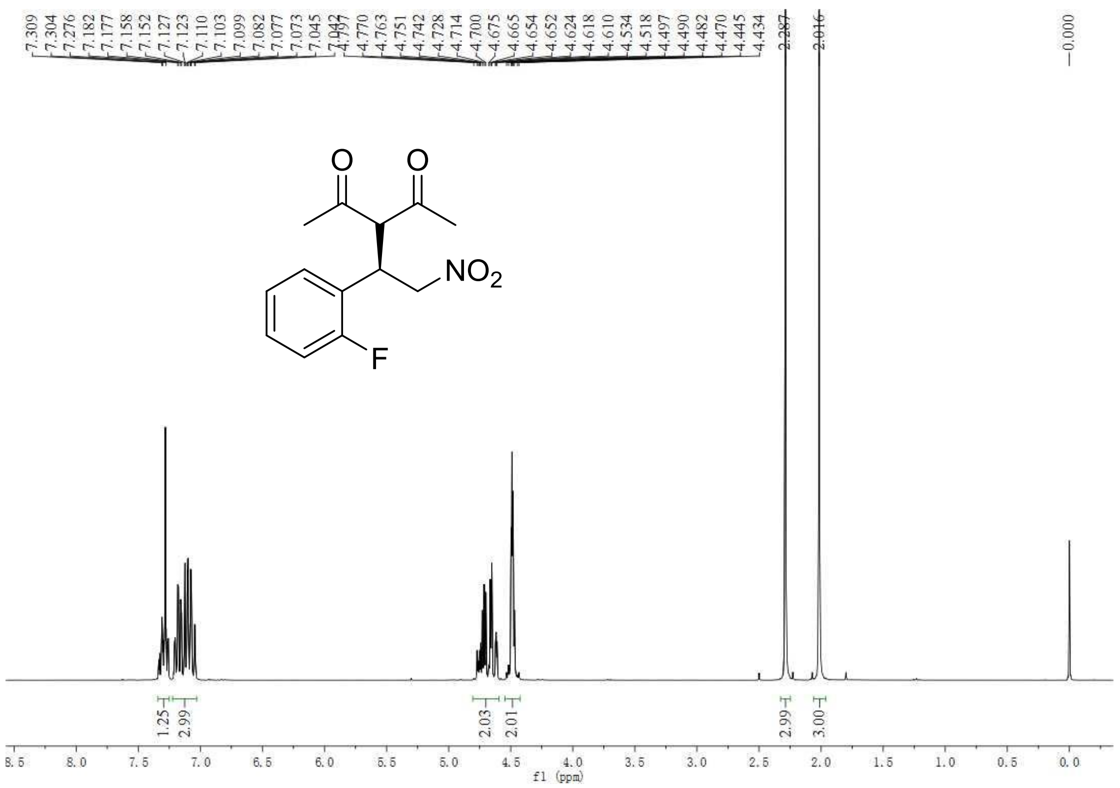

^{13}C NMR spectrum of **9i**

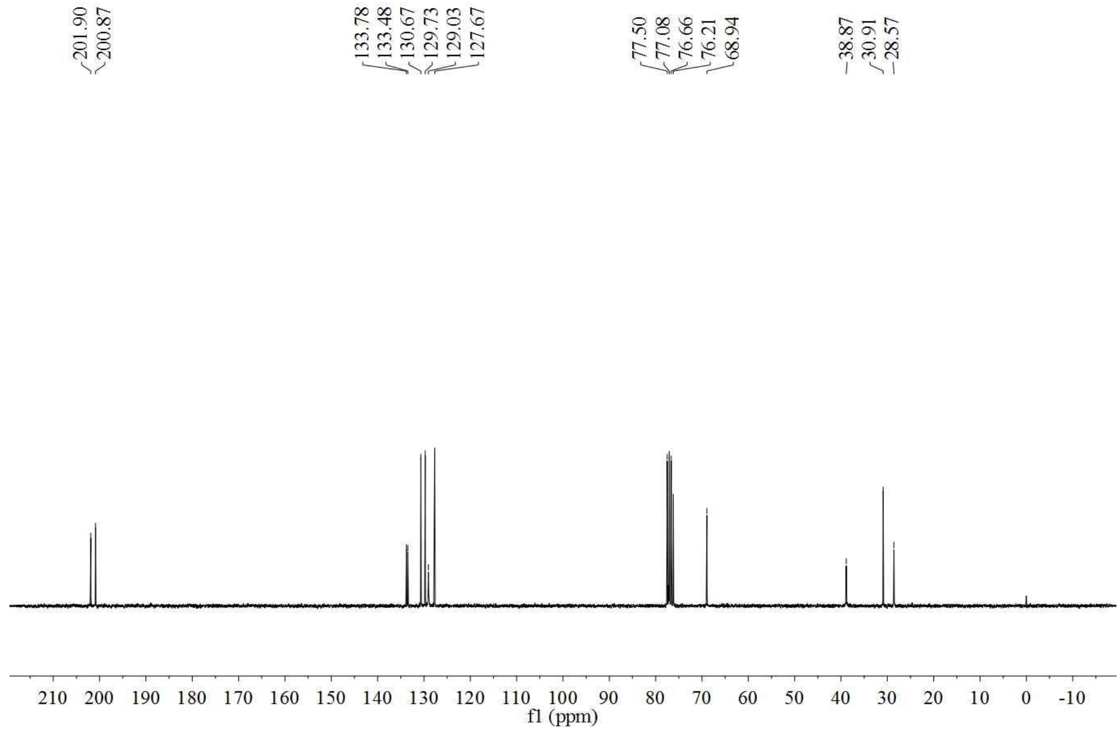

^{1}H NMR spectrum of **9j**

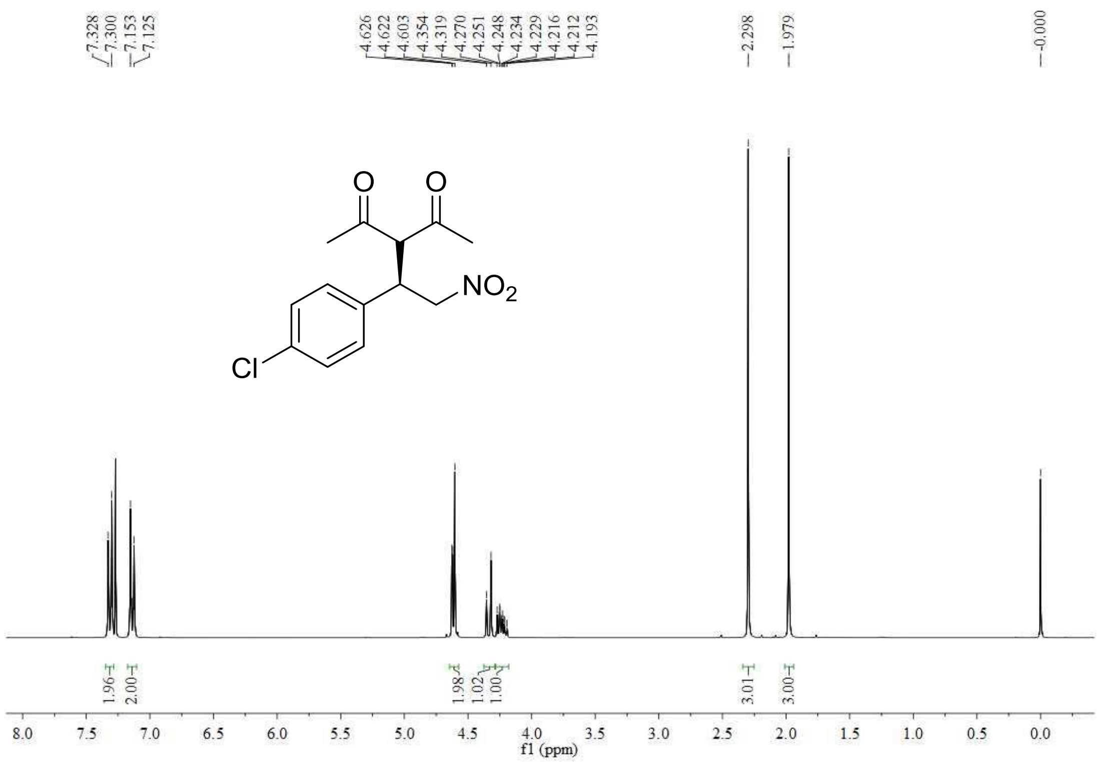

^{13}C NMR spectrum of **9j**

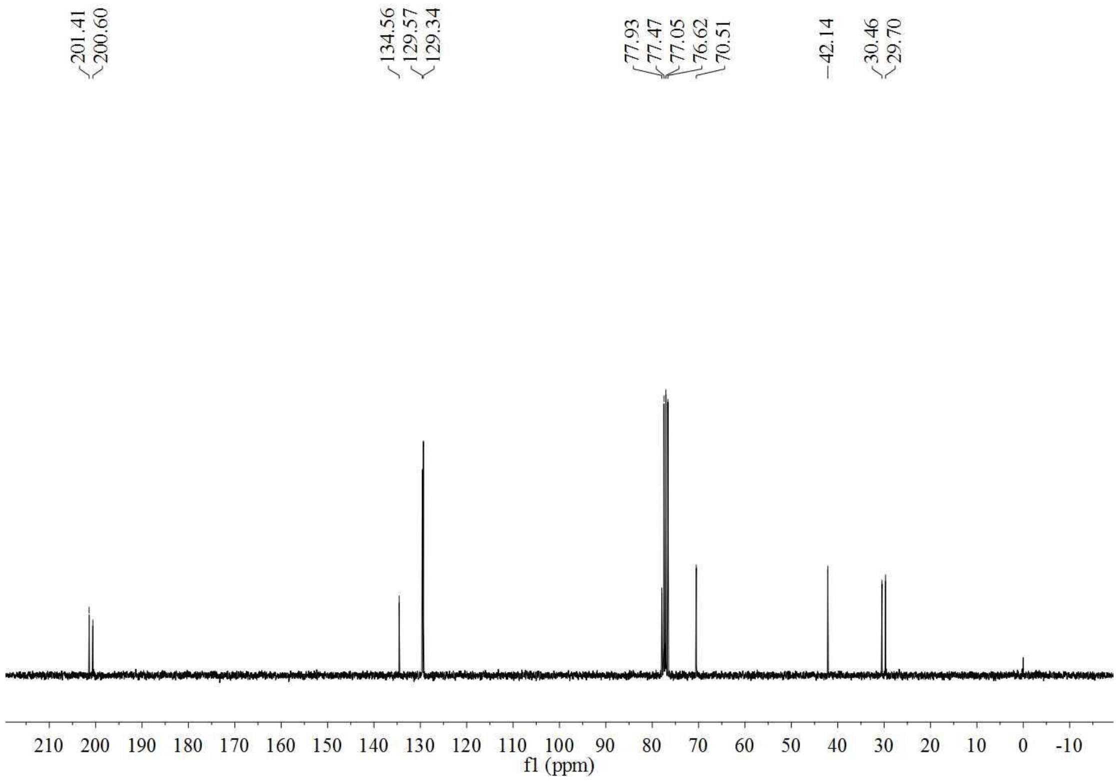

^{1}H NMR spectrum of **9k**

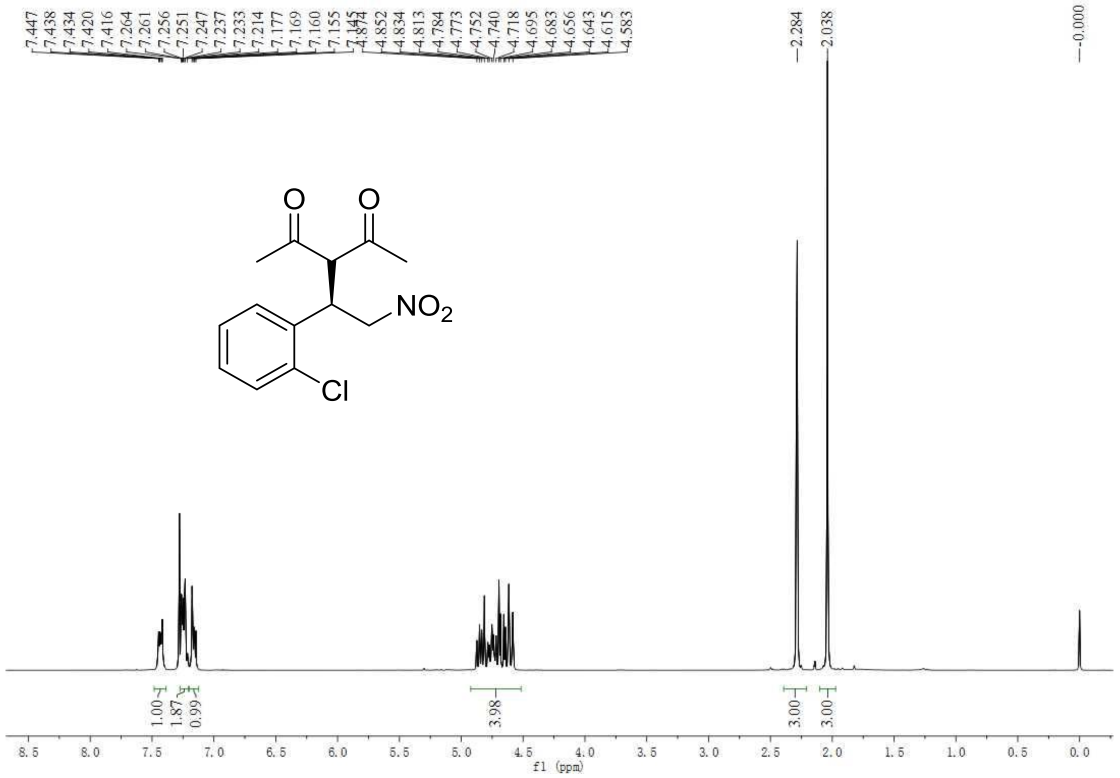

^{13}C NMR spectrum of **9k**

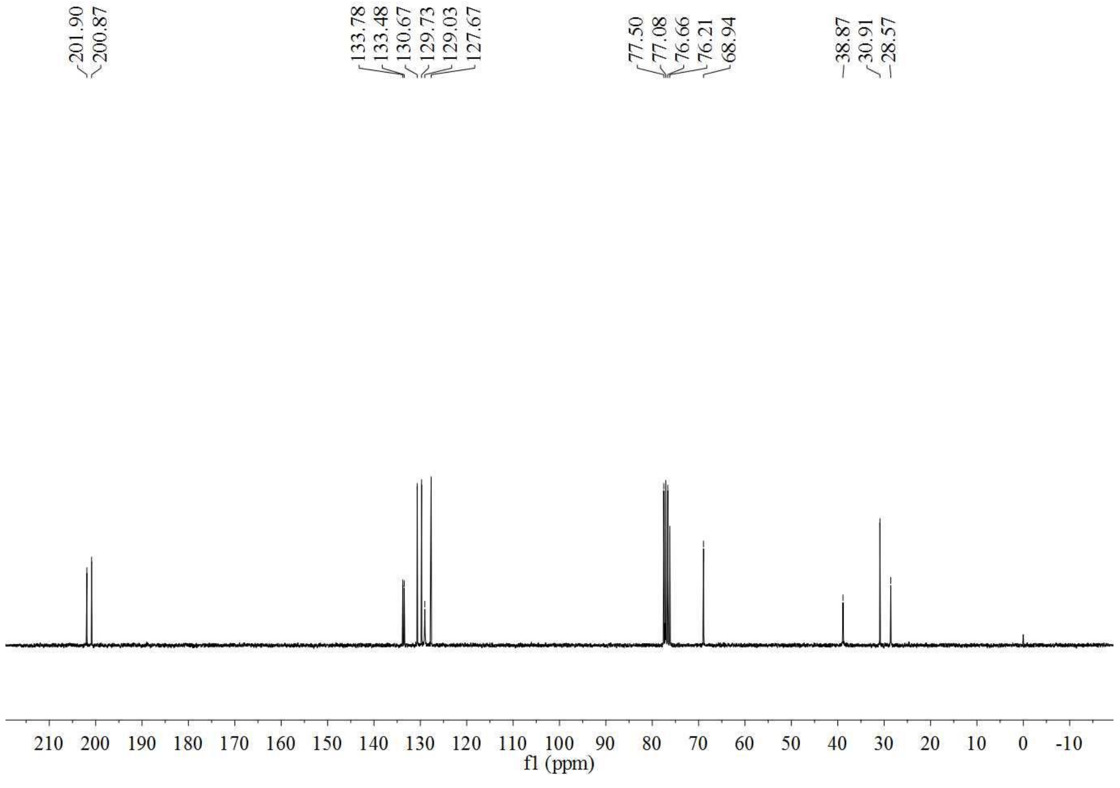

6. HPLC chromatograms

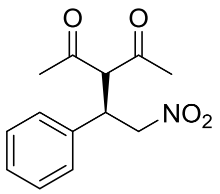

9a

(Racemic)

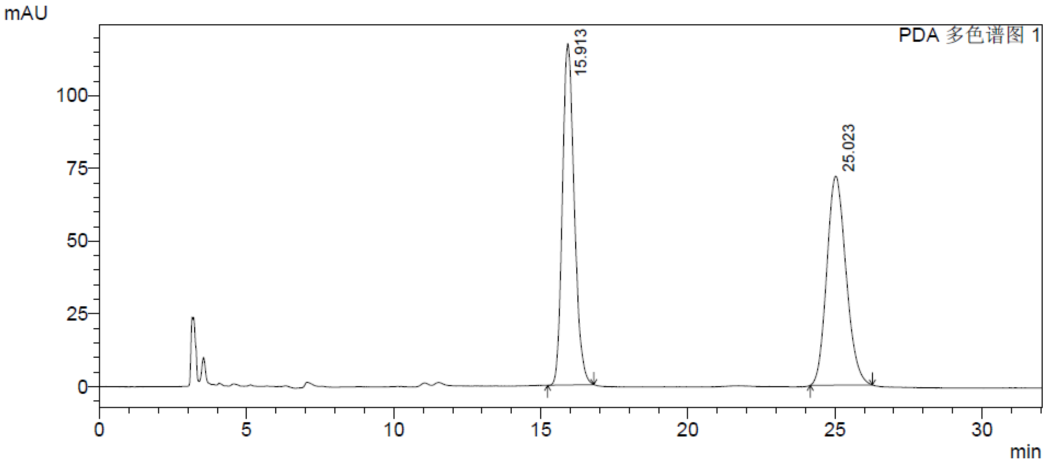

(Chiral)

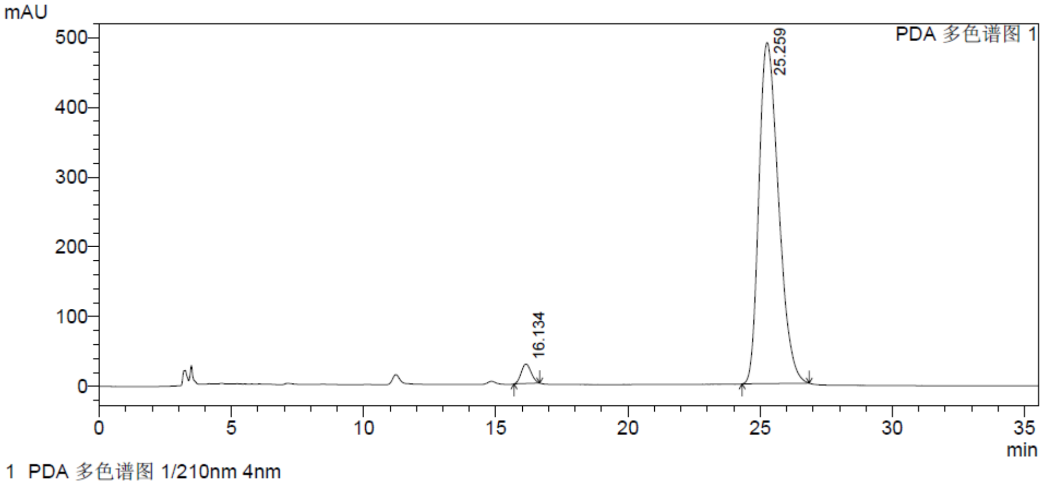

1 PDA 多色谱图 1/210nm 4nm

峰表

PDA Ch1 210nm 4nm

峰#	保留时间	面积	高度	面积 %	高度 %
1	16. 134	728965	27922	2. 803	5. 401
2	25. 259	25282071	489071	97. 197	94. 599
总计		26011036	516993	100. 000	100. 000

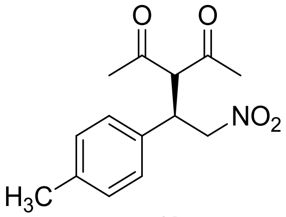

9b

(Racemic)

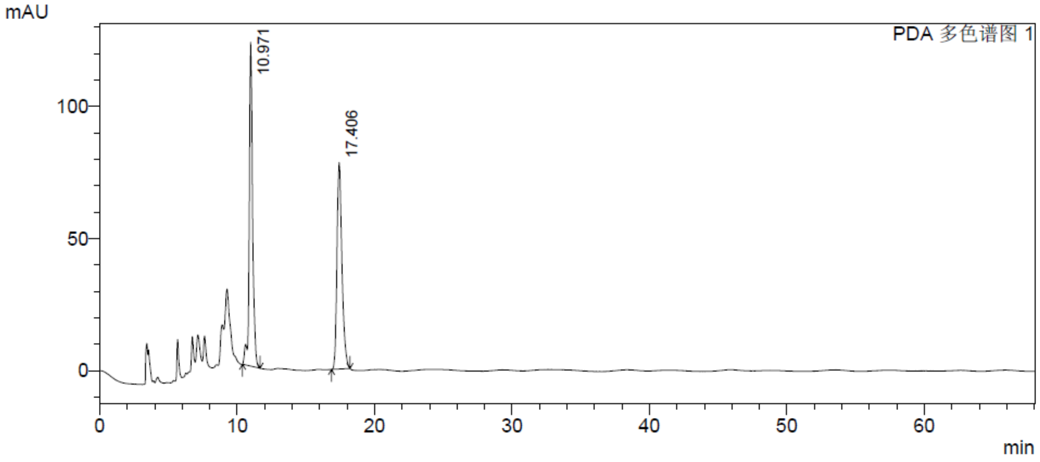

(Chiral)

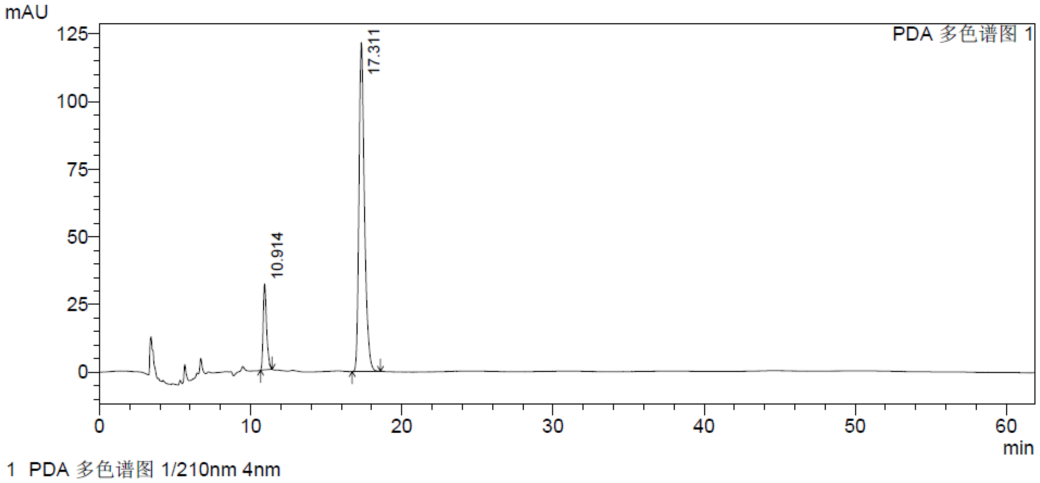

1 PDA 多色谱图 1/210nm 4nm

峰表

PDA Ch1 210nm 4nm

峰#	保留时间	面积	高度	面积 %	高度 %
1	10.914	500672	31766	13.878	20.716
2	17.311	3107098	121578	86.122	79.284
总计		3607770	153344	100.000	100.000

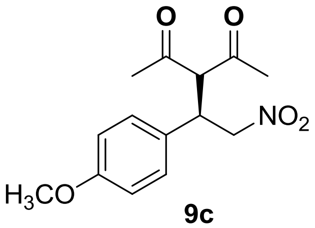

9c

(Racemic)

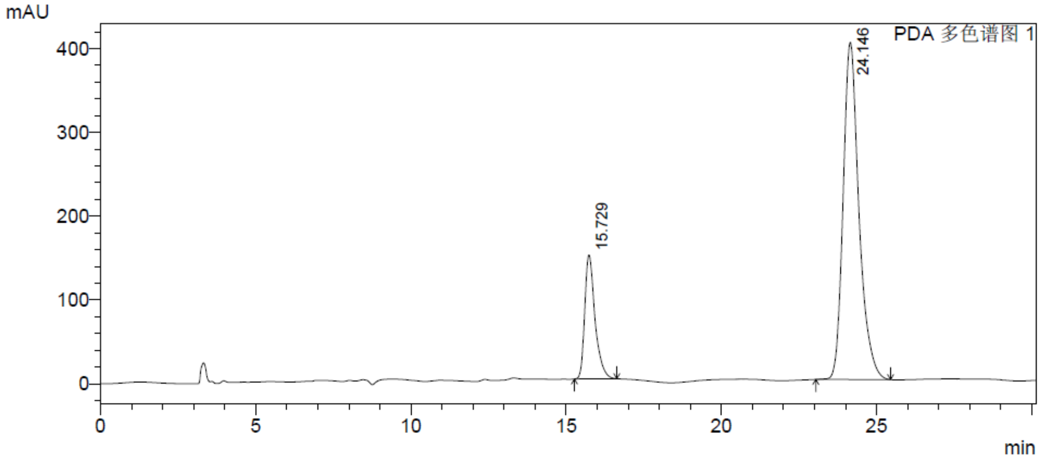

(Chiral)

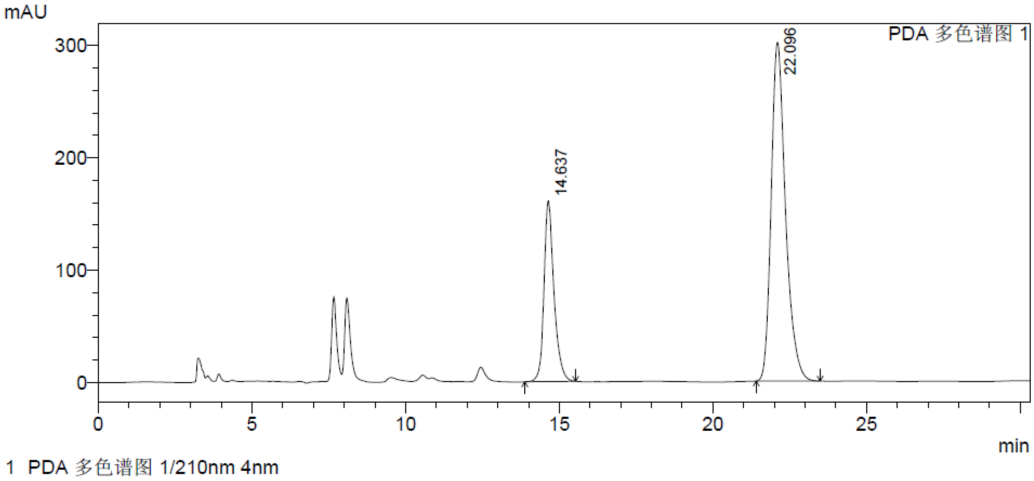

1 PDA 多色谱图 1/210nm 4nm

峰表

PDA Ch1 210nm 4nm

峰#	保留时间	面积	高度	面积 %	高度 %
1	14.637	3639831	161120	27.125	34.815
2	22.096	9779132	301673	72.875	65.185
总计		13418963	462793	100.000	100.000

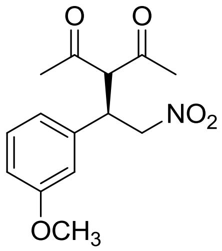

9d

(Racemic)

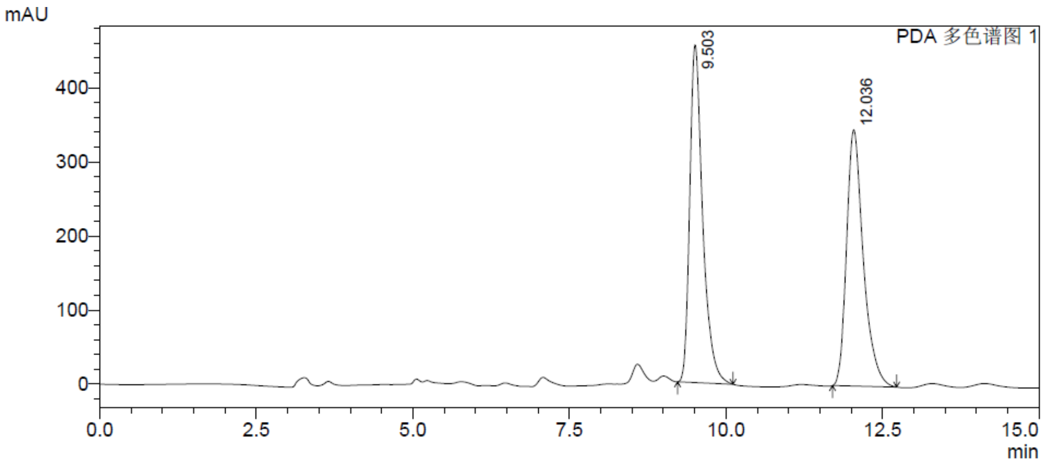

(Chiral)

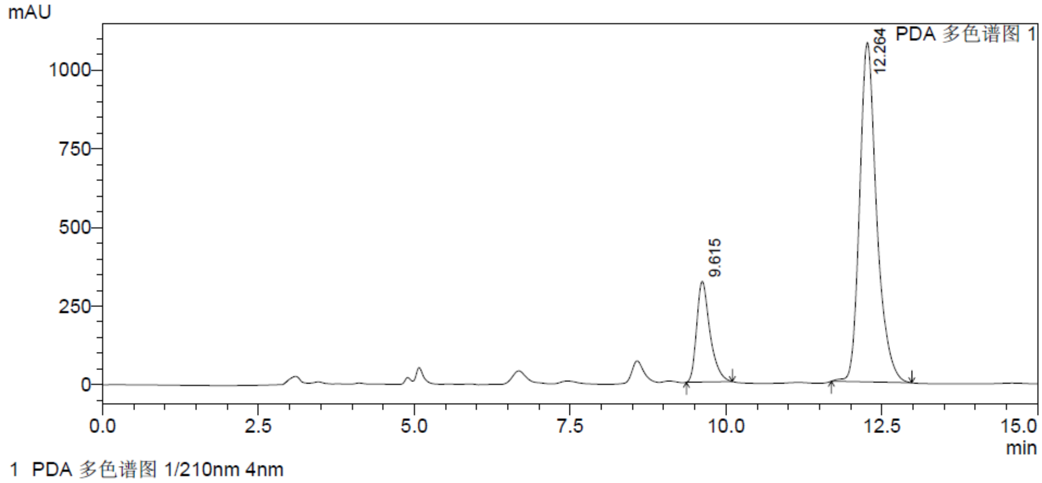

1 PDA 多色谱图 1/210nm 4nm

峰表

PDA Ch1 210nm 4nm

峰#	保留时间	面积	高度	面积 %	高度 %
1	9.615	4735510	320163	18.679	22.867
2	12.264	20616832	1079926	81.321	77.133
总计		25352342	1400089	100.000	100.000

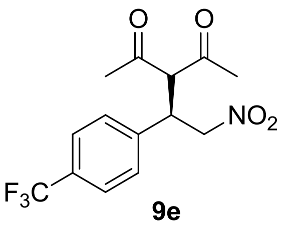

9e

(Racemic)

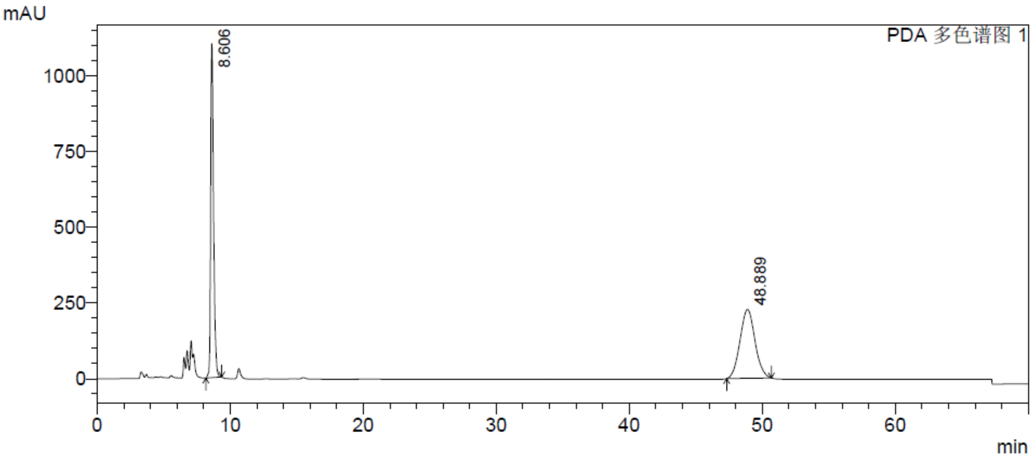

(Chiral)

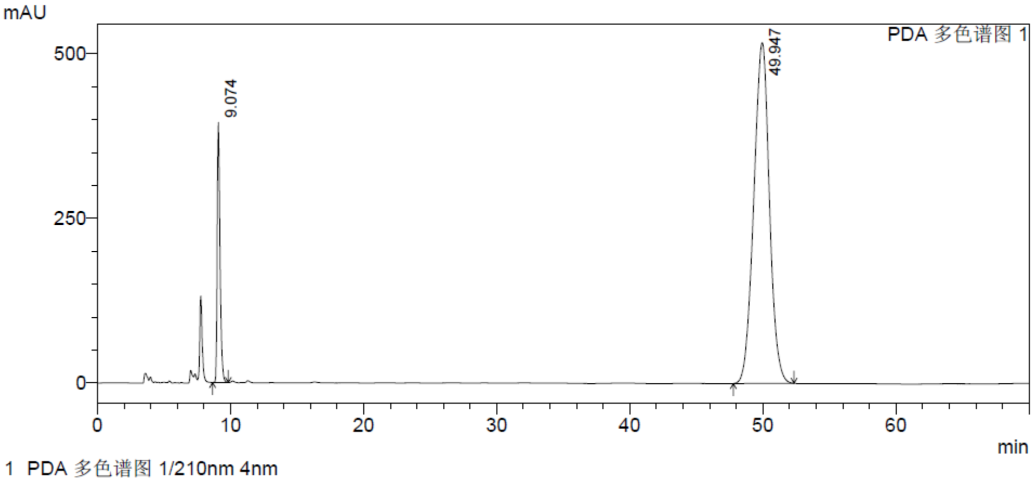

1 PDA 多色谱图 1/210nm 4nm

峰表

PDA Ch1 210nm 4nm

峰#	保留时间	面积	高度	面积 %	高度 %
1	9.074	5809439	395093	12.060	43.290
2	49.947	42360578	517567	87.940	56.710
总计		48170016	912659	100.000	100.000

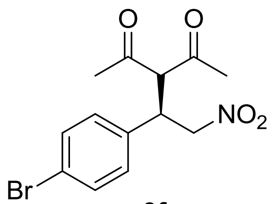

9f

(Racemic)

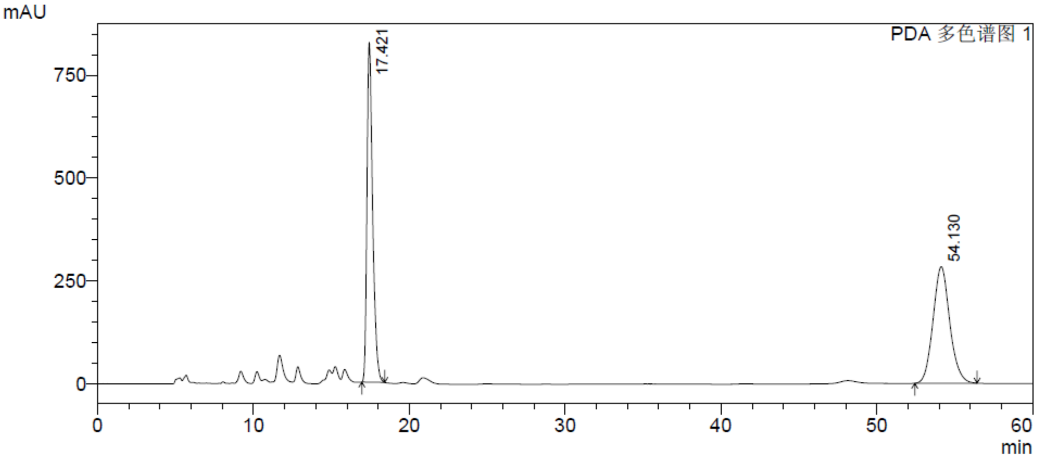

(Chiral)

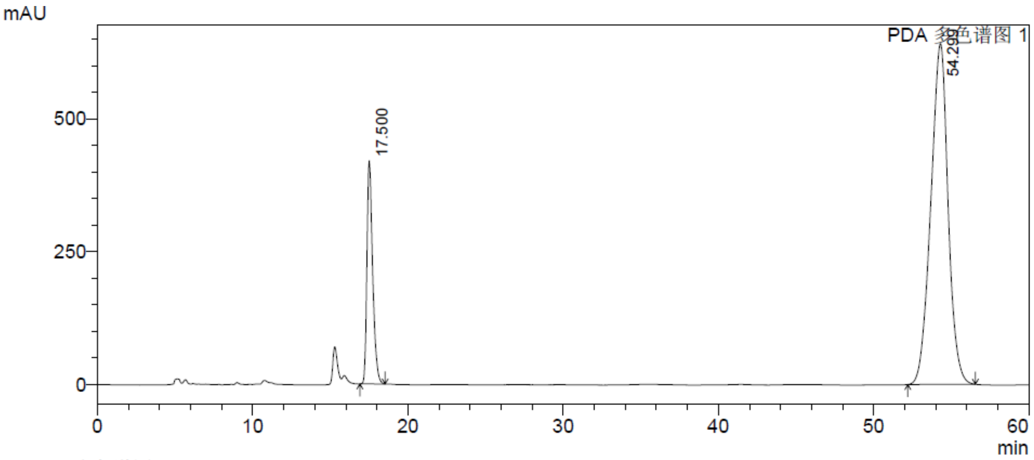

1 PDA 多色谱图 1/210nm 4nm

峰表

PDA Ch1 210nm 4nm

峰#	保留时间	面积	高度	面积 %	高度 %
1	17. 500	10770979	420431	17. 834	39. 571
2	54. 299	49624893	642041	82. 166	60. 429
总计		60395872	1062472	100. 000	100. 000

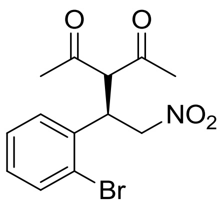

9g

(Racemic)

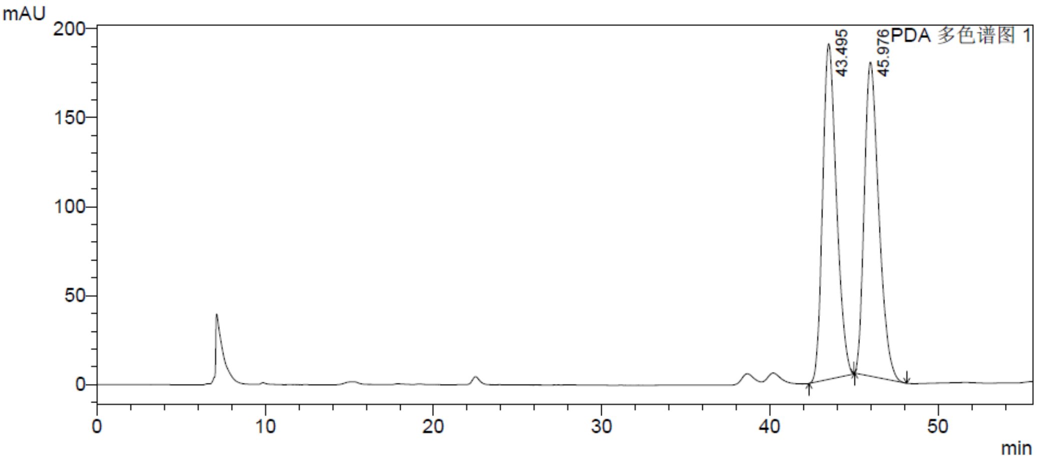

(Chiral)

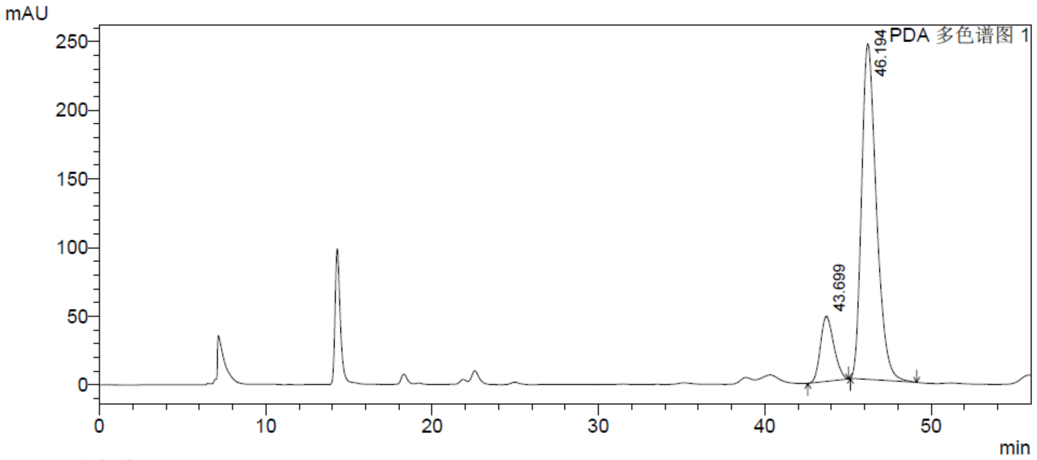

1 PDA 多色谱图 1/210nm 4nm

峰表

PDA Ch1 210nm 4nm

峰#	保留时间	面积	高度	面积 %	高度 %
1	43.699	2648025	47438	14.857	16.254
2	46.194	15174997	244416	85.143	83.746
总计		17823023	291854	100.000	100.000

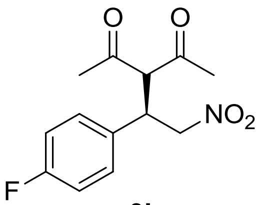

9h

(Racemic)

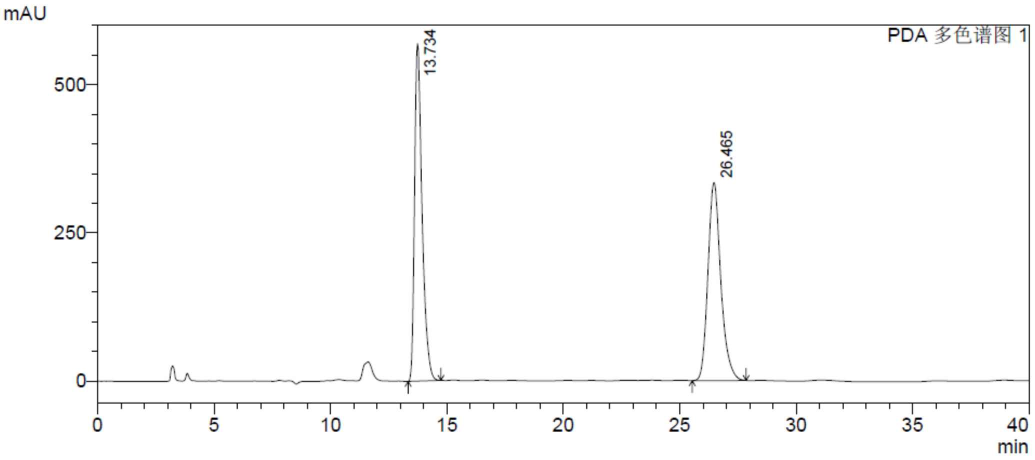

(Chiral)

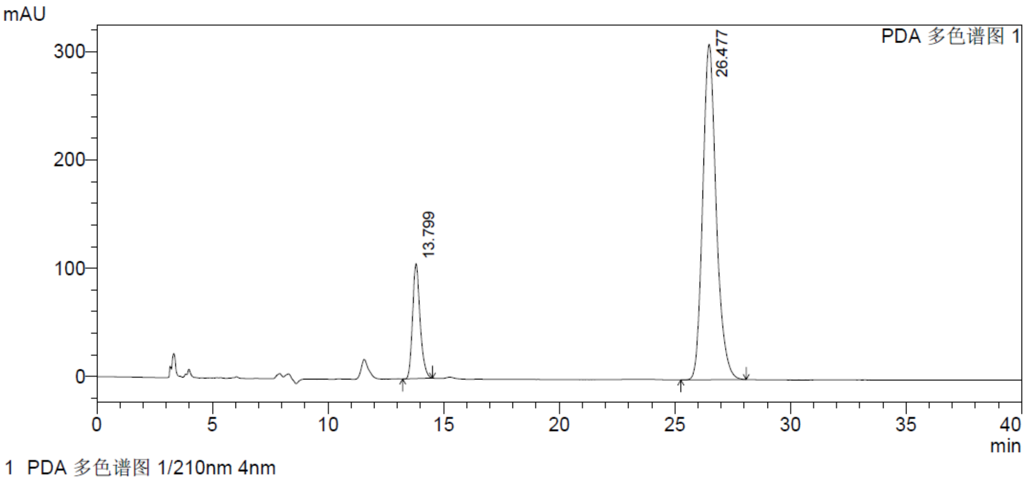

峰表

PDA Ch1 210nm 4nm

峰#	保留时间	面积	高度	面积 %	高度 %
1	13.799	2401325	106232	15.999	25.537
2	26.477	12608096	309763	84.001	74.463
总计		15009422	415995	100.000	100.000

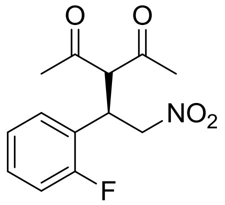

9i

(Racemic)

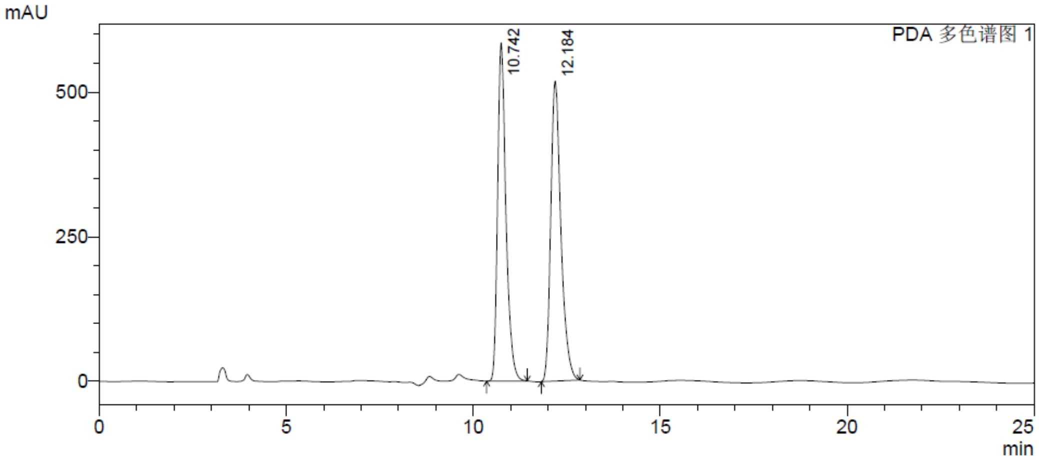

(Chiral)

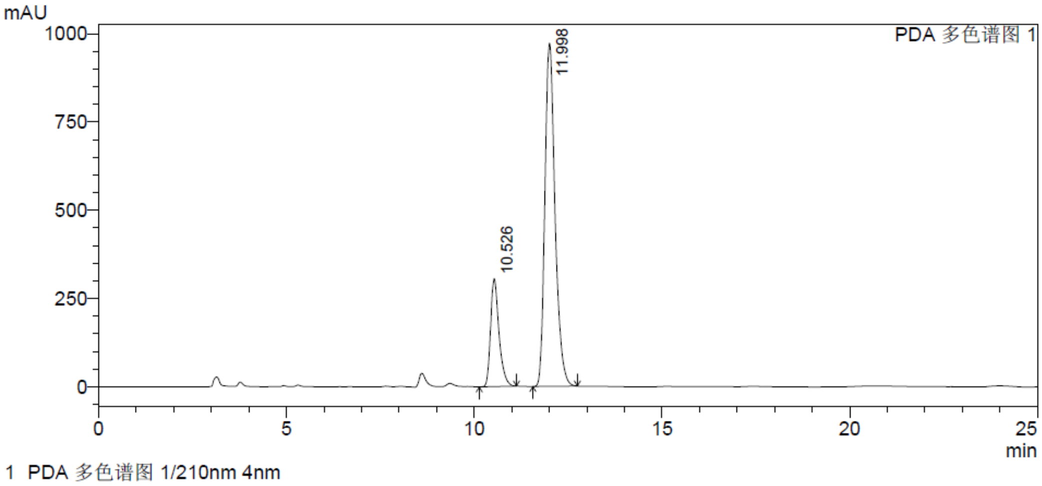

1 PDA 多色谱图 1/210nm 4nm

峰表

PDA Ch1 210nm 4nm

峰#	保留时间	面积	高度	面积 %	高度 %
1	10.526	4765876	305868	20.650	23.933
2	11.998	18313815	972140	79.350	76.067
总计		23079691	1278008	100.000	100.000

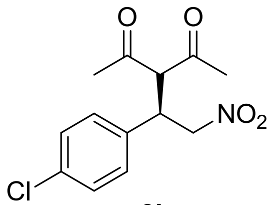

9j

(Racemic)

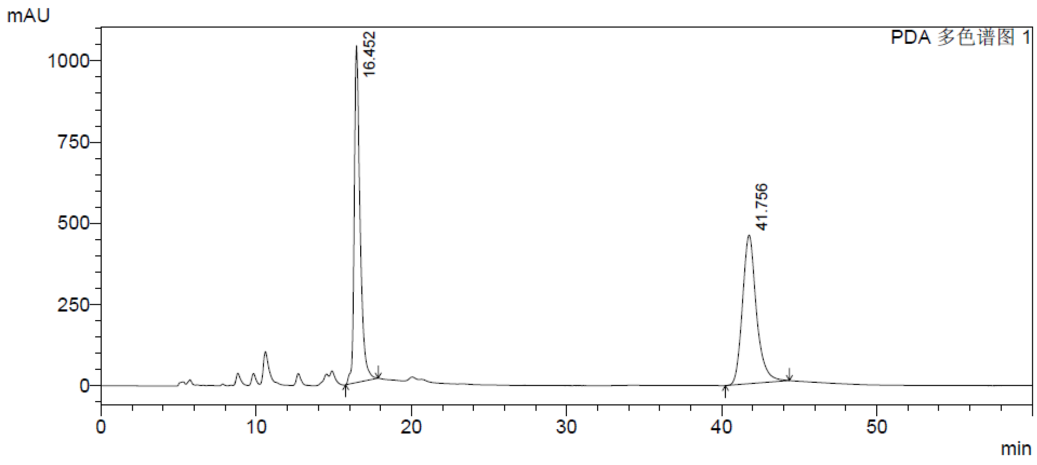

(Chiral)

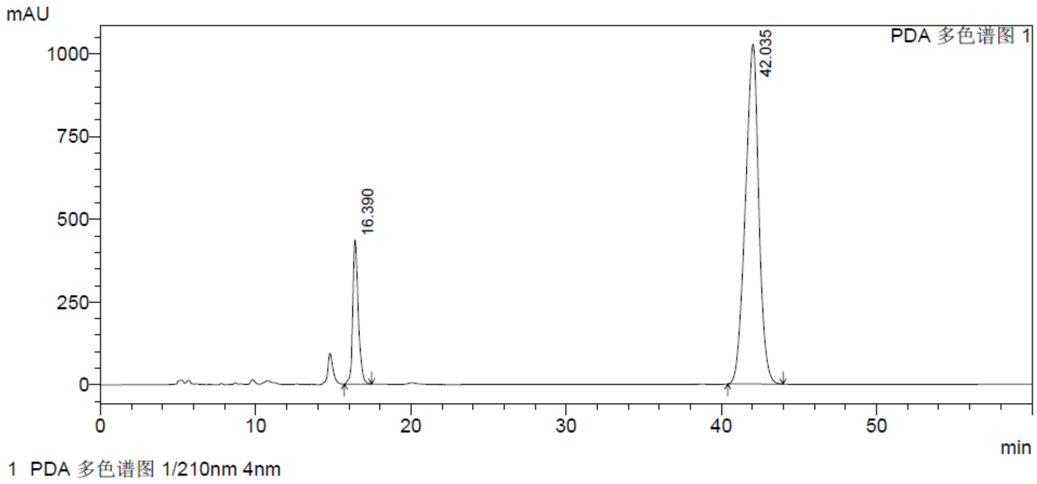

峰表

PDA Ch1 210nm 4nm

峰#	保留时间	面积	高度	面积 %	高度 %
1	16.390	10772952	435053	14.824	29.753
2	42.035	61899906	1027173	85.176	70.247
总计		72672858	1462226	100.000	100.000

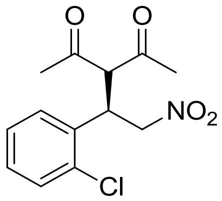

9k

(Racemic)

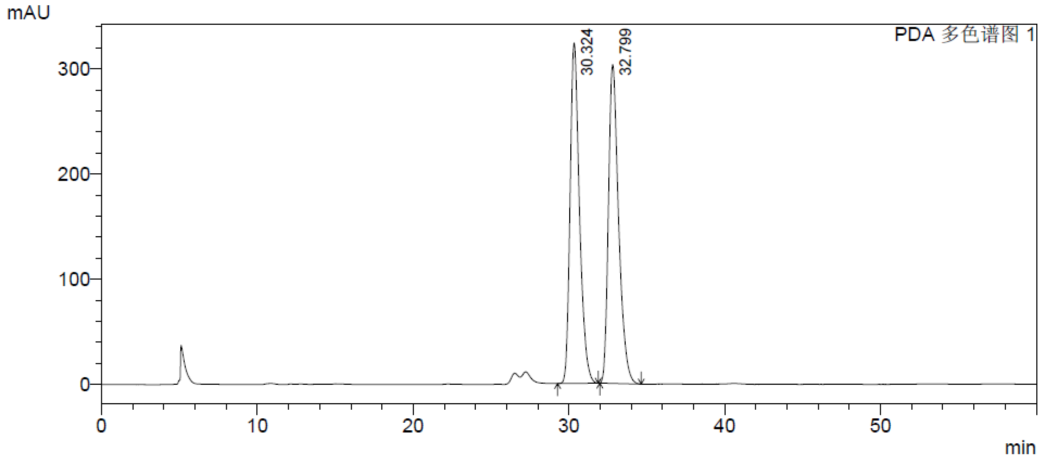

(Chiral)

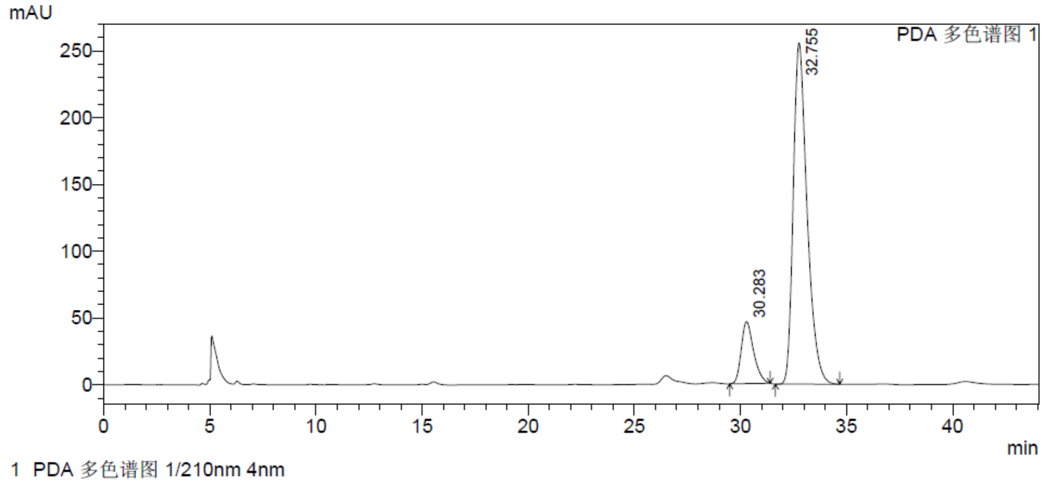

1 PDA 多色谱图 1/210nm 4nm

峰表

PDA Ch1 210nm 4nm

峰#	保留时间	面积	高度	面积 %	高度 %
1	30.283	1879169	46314	13.942	15.340
2	32.755	11599093	255600	86.058	84.660
总计		13478262	301914	100.000	100.000

7. References

1. Amarasinghe, N. R.; Turner, P.; Todd, M. H. *Adv. Synth. Catal.* **2012**, *354*, 2954-2958.

2. Li, Z. Y.; Ma, J. J.; Chen, J. W.; Pan, Y.; Jiang, J. L.; Wang, L. Y. *Chinese J. Chem.* **2009**, *27*, 2031-2036.

3. Durmaz, M.; Tataroglu, A.; Yilmaz, H.; Sirit, A. *Tetrahedron-Asymmetr* **2016**, *27*, 148-156.

4. Dong, Z.; Qiu, G. F.; Zhou, H. B.; Dong, C. N. *Tetrahedron-Asymmetr* **2012**, *23*, 1550-1556.

5. Ren, X.; He, C.; Feng, Y.; Chai, Y.; Yao, W.; Chen, W.; Zhang, S. *Org. Biomol. Chem.* **2015**, *13*, 5054-5060.
